# Reducing bias in RNA sequencing data: a novel approach to compute counts

**DOI:** 10.1186/1471-2105-15-S1-S7

**Published:** 2014-01-10

**Authors:** Francesca Finotello, Enrico Lavezzo, Luca Bianco, Luisa Barzon, Paolo Mazzon, Paolo Fontana, Stefano Toppo, Barbara Di Camillo

**Affiliations:** 1 Department of Information Engineering, University of Padova, Padova, 35131, Italy; 2 Department of Molecular Medicine, University of Padova, Padova, 35131, Italy; 3 Research and Innovation Centre, Edmund Mach Foundation, Trento, 38010, Italy

## Abstract

**Background:**

In the last decade, Next-Generation Sequencing technologies have been extensively applied to quantitative transcriptomics, making RNA sequencing a valuable alternative to microarrays for measuring and comparing gene transcription levels. Although several methods have been proposed to provide an unbiased estimate of transcript abundances through data normalization, all of them are based on an initial count of the total number of reads mapping on each transcript. This procedure, in principle robust to random noise, is actually error-prone if reads are not uniformly distributed along sequences, as happens indeed due to sequencing errors and ambiguity in read mapping.

Here we propose a new approach, called *maxcounts*, to quantify the expression assigned to an exon as the maximum of its per-base counts, and we assess its performance in comparison with the standard approach described above, which considers the total number of reads aligned to an exon. The two measures are compared using multiple data sets and considering several evaluation criteria: independence from gene-specific covariates, such as exon length and GC-content, accuracy and precision in the quantification of true concentrations and robustness of measurements to variations of alignments quality.

**Results:**

Both measures show high accuracy and low dependency on GC-content. However, *maxcounts *expression quantification is less biased towards long exons with respect to the standard approach. Moreover, it shows lower technical variability at low expressions and is more robust to variations in the quality of alignments.

**Conclusions:**

In summary, we confirm that counts computed with the standard approach depend on the length of the feature they are summarized on, and are sensitive to the non-uniform distribution of reads along transcripts. On the opposite, *maxcounts *are robust to biases due to the non-uniformity distribution of reads and are characterized by a lower technical variability. Hence, we propose *maxcounts *as an alternative approach for quantitative RNA-sequencing applications.

## Background

In recent years, ultra-high-throughput sequencing technologies (also called *Next-Generation Sequencing *technologies, NGS) [1,2] have been applied intensively in quantitative transcriptomics, making RNA sequencing (RNA-seq) [3] a valuable alternative to microarrays. While microarrays can only assay transcripts corresponding to probes, RNA-seq can, in principle, investigate at a finer level of detail all the transcripts present in a sample, characterizing their sequences and quantifying their abundances at the same time [4]. The possibility of sequencing transcriptomes at single-base resolution has opened a wide frontier of applications in transcriptomics research: transcriptome profiling of non-model organisms [5,6], novel transcripts discovery [7], quantification of allele-specific gene expression [8], investigation of RNA editing [9,10] and "dual RNA-seq" of pathogen and host [11]. In this work we focus on its application to quantitative transcriptomics, since RNA-seq is now widely used in place of microarrays for measuring and comparing gene transcription levels [4,12]. The standard workflow of transcripts quantification with RNA-seq is the following: first, RNAs are extracted from the sample of interest and subjected to fragmentation; then, RNA fragments are reverse-transcribed into complementary DNAs (cDNAs); finally, cDNAs are ligated to adapters and subjected to ultra-high-throughput sequencing. The millions of short sequences generated, called *reads*, can be aligned to a reference genome or transcriptome to calculate *counts *(i.e. the number of reads aligned to each gene or transcript), which give a *digital *measure of transcript abundances in the original sample. However, this measure requires normalization to correct for systematic errors arising from several sources of bias. First of all, the largest fraction of the reads sequenced in a sample arises from a restricted subset of highly expressed genes [13,14]; as a consequence, these genes account for most of the counts in a library, while the remaining genes are under-represented. Moreover, by definition, counts are intrinsically biased towards longer transcripts: longer transcripts are more likely to be sequenced than shorter ones, so counts depend not only on the true gene expression, but also on the length of transcribed isoforms [15-19]. In addition, recent works highlight other sequence-dependent sources of bias affecting NGS data [20-23]. In particular, many studies observe the presence of a GC-content effect: gene counts correlate with the fraction of "G" (guanine) and "C" (cytosine) bases in the nucleotidic sequence of a gene [23-25].

Although several methods have been proposed to normalize data, thus providing less biased estimates of transcript abundances, all of them are based on an initial count of the total number of reads mapping on each transcript [19,24-26]. This procedure, in principle robust to random noise, might be error-prone if reads are not uniformly distributed along sequences, as happens indeed due to both sequencing errors and ambiguity in read mapping.

Non-uniformity of read coverage is mainly due to biases associated to the different steps of RNA-seq protocols. For instance, fragmentation methods based on restriction enzymes have recently been reported to be sequence-specific and far from being random [27]. Reverse-transcription performed with poly-dT oligomers, which bind to poly-A tails, is strongly biased towards 3' end of transcripts [3,4]. Conversely, reverse-transcription with random hexamers results in an under-representation of 3' ends [4,27]. This bias is due to the reduced number of priming positions from which the reverse transcriptase enzyme can start cDNA synthesis. Furthermore, depending on their sequence, RNAs and cDNAs can form secondary structures that alternatively obstruct or facilitate the binding of reverse-transcription primers and sequencing adapters, resulting in different efficiency of the sequencing process [28]. Since the first RNA-seq experiment [3], several changes in library preparations and sequencing protocols have been proposed pursuing the aim of having an unbiased representation of transcript abundances (e.g. postponing reverse transcription after fragmentation), but the non-uniformity of read coverage along transcripts remains an issue of state-of-the-art technologies [29].

In this study, we propose a novel method for computing counts, called *maxcounts*, with the aim of reducing systematic errors. Once reads have been aligned to a feature of interest (exon or single-isoform transcript), we exploit read coverage to obtain counts for every position in its sequence and we quantify its expression as the maximum of its "positional" counts. We assess *maxcounts *performance in comparison with the standard approach, which considers the total number reads mapped on an exon (called *totcounts *in the following). To do this we considered three human data sets [19,30,31], in which samples are taken from different tissues or cellular compartments, or from cells subjected to different growth conditions or treatments. All samples were sequenced with the Illumina technology (http://www.illumina.com), which is now the most commonly used NGS platform for RNA-seq [32]. Data were sequenced with single- and paired-end protocols, and have different characteristics, which allow us to test our approach with respect to different features. In particular, in Jiang's experiment [31], endogenous RNAs were sequenced together with spike-in RNAs, which are single-isoform transcripts with known nucleotidic sequences and concentrations. We used these data as gold-standard to benchmark and compare *totcounts *and *maxcounts *estimates of RNA abundances.

## Methods

### Data sets

The MAQC2 data set [19] consists of single-ended RNA-seq reads obtained from two different biological samples: (i) Ambion's Human Brain Reference RNA ("Brain"), a standard pooled from multiple donors and several brain regions; (ii) Stratagene's Universal Human Reference RNA ("UHR"), a mixture of total RNA extracted from ten different human cell lines (see "Additional file 1" for further details on data).

Griffith's data set [30] contains paired-end reads obtained sequencing two fluorouracil (5-FU)-resistant ("MIP5FU") and (5-FU)-sensitive ("MIP101") human colorectal cancer cell lines.

A subset of replicates from Jiang's data set [31] is also considered, in which paired-end RNA-seq libraries were sequenced after mixing endogenous RNA from a K-562 cell line, extracted from nucleus ("nucleus") or whole cell ("cell"), with RNA standards developed by the External RNA Control Consortium (ERCC). ERCC standards are in vitro synthesized RNAs whose nucleotidic sequences and concentrations are known. They can be used to assess whether the final quantification of an RNA-seq experiment correctly represents the composition of the original input.

### Pre-processing analysis pipeline

**Figure 1:**
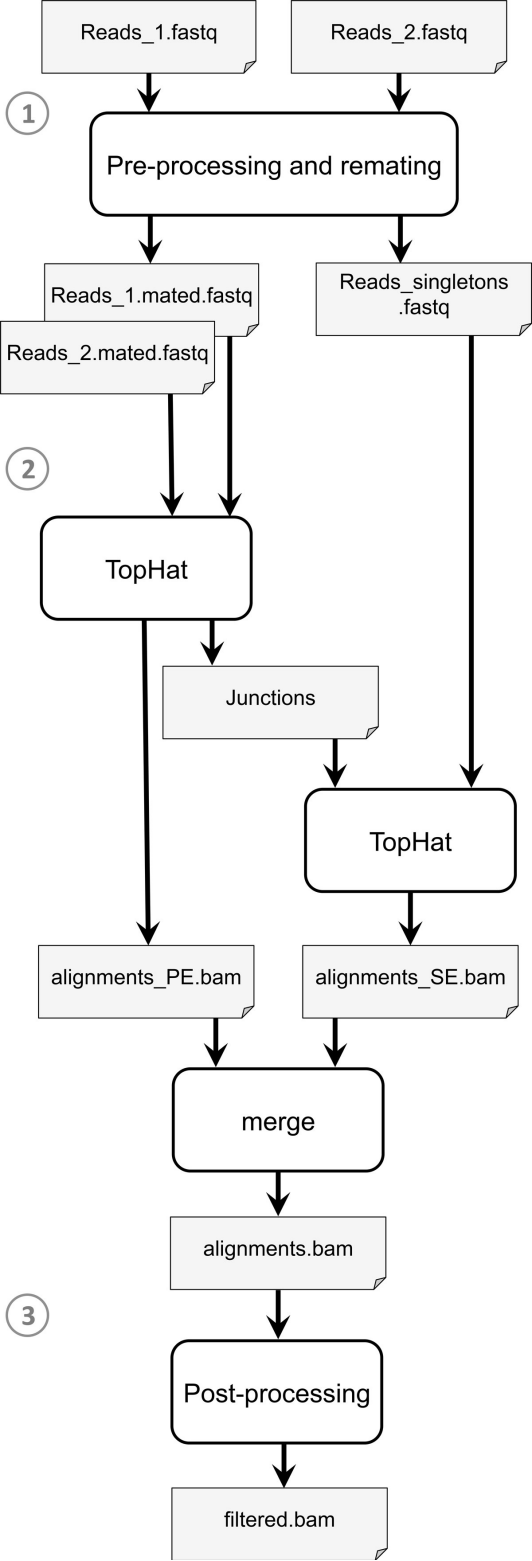
**Analysis pipeline**. 1) Read pre-processing (trimming and filtering) and re-mating of paired-ends. 2) Separate mapping of paired-end and singleton reads, and merging of alignments. 3) Removal of low similarity alignments and multireads.

We defined and implemented a pipeline to pre-process and map reads, and to discard low-similarity alignments and *multireads *(i.e. reads mapping to multiple positions of the reference). The analysis pipeline implemented in this study is depicted by the flowchart of Figure 1 (see "Additional file 1" for further details on pipeline implementation). A simplified version of the same pipeline was applied to single-end data.

In the first phase, reads were pre-processed to remove adapter sequences and read ends whose Phred quality was lower than 20, and to discard reads whose length after trimming was less than 33 bp. Output FASTQ files were re-formatted to recover the correspondence of paired-end reads, and to store in a separate file the singleton reads, whose mate was discarded during pre-processing.

In the second phase, paired-ends and singletons were mapped with TopHat [33] in a two-steps procedure. First, paired-end reads were mapped on the reference sequence to generate a BAM file of alignments and a file of junctions. Then, singletons were mapped with TopHat exploiting the information provided by junctions (see "Additional file 1" for further details on read mapping). Alignment files from paired-end and singleton reads were finally merged in a single BAM file using the merge utility of samtools [34].

In the last phase of post-processing, a filtered set of alignments was obtained after discarding multireads and reads whose similarity with the reference was lower than 97%. This analysis was performed using SAMsieve, a java in-house developed program (available upon request), which allows the user to filter alignments stored in SAM or BAM files based on several criteria (see "Additional file 1" for additional information about SAMsieve).

### Computation of counts and normalization

*Totcounts *were computed using bedtools [35]. Exons (or spike-in transcripts of Jiang's data set) with average *totcounts *across replicates lower than 0.5 were discarded from our analysis. Before comparing or averaging replicates, differences in library sizes were corrected through Trimmed Mean of M-values (TMM) normalization [13].

For each exon i in library j, *maxcounts *were computed as:

$$maxcounts_{\text{ij}}=\max\left({\text{N}}_{\text{ijp}}\right)$$

where, N_ijp _is the number of reads covering position p along the exon. We implemented the method for computing *maxcounts *in a new patch for bedtools that can be downloaded from http://www.dei.unipd.it/~finotell/maxcounts/ (see additional details in "Additional file 1"). Also in this case, exons with average *maxcounts *across replicates lower than 0.5, were discarded and differences in library sizes were corrected with TMM normalization. In the following, we will refer to TMM-normalized *totcounts *and *maxcounts *simply as "*totcounts*" and "*maxcounts*". Although providing an assessment of normalization methods is beyond the scope of the present work, we acknowledge that length bias can be corrected trough normalization. Thus, to guarantee a fair comparison with current standards, we applied, when necessary, two normalization approaches: *Reads Per Kilobase of exon Model per million mapped reads *(RPKM) [16], which is widely used in RNA-seq studies, and within-lane full-quantile normalization, using exon length as covariate, since it has been proposed as preferred method in a recent work by Risso *et al. *[24]. RPKMs for each exon i in library j were calculated as follows:

RPKMij=NijLi103⋅N⋅j106

where, N_ij _are counts for exon i in library j (not normalized *via *TMM), L_i _is the length of exon i and N._j _= ∑_i _N_ij _is the sum of all counts in library j.

Within-lane full-quantile normalization of counts on exon length was performed using EDASeq [24]. In order to correct for differences in library sizes, this normalization was used together with between-lane full-quantile normalization, also implemented in EDASeq.

In this work we consider exons instead of genes or transcripts as we intend to evaluate the different summarization methods described above without biases, possibly introduced by the choice of a transcription model (e.g. how overlapping genes or alternative spliced exons are considered).

## Results and discussion

Ideally, a measure of gene expression should: (i) be independent of gene-specific covariates such as transcript length and GC-content; (ii) be unbiased towards highly expressed genes; *(iii) *be an accurate estimate of the true gene expression levels; *(iv) *show low technical variance; (*v*) be robust to possible variations in the quality of alignments. In the following we assess the above properties for *maxcounts *in comparison with *totcounts*. Plots are shown for Jiang's data, since this data set allows also the assessment of accuracy in transcript quantification thanks to spike-in RNAs; results on MAQC2 and Griffith's are reported in Additional Files.

### Length and GC-content biases

**Figure 2:**
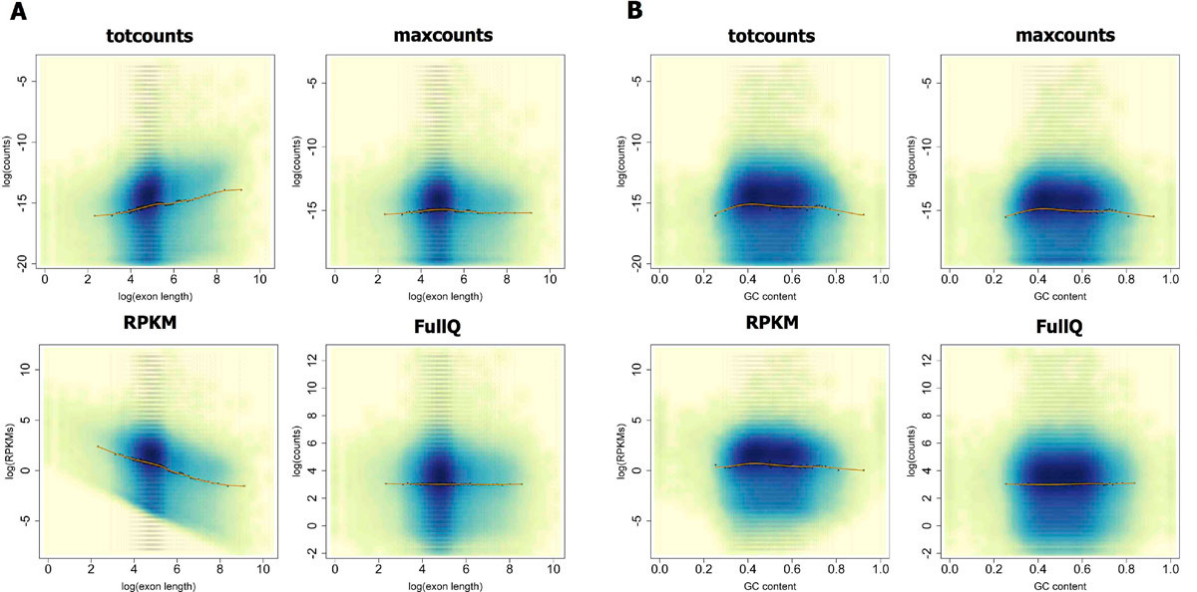
**Exon length bias and GC-content effect**. Smoothed scatter-plots showing the relationship between log-counts/RPKMs and exon length (log scale, **A**) or GC-content (**B**), in Jiang's data ("nucleus" libraries). The orange curve represents a cubic-spline fit computed on log-counts, averaged in bins of 5000 exons each (black crosses between vertical lines, indicating bin bounds). Counts or RPKMs are computed using *totcounts*, *maxcounts*, RPKM-corrected *totcounts *(*RPKM*) and *totcounts *corrected with within-lane full-quantile normalization over exon length (*FullQ*), and averaged across libraries.

To investigate exon length bias, we used smoothed scatter-plots of counts (averaged across replicates) versus exon-length (Figure 2A and Additional Files 2, 3, 4, 5, 6).

In all data sets, plots show an increasing trend of *totcounts *as exon length increases (see the cubic-spline fit represented by the orange line), revealing that longer exons tend to have higher counts than shorter ones. This bias is reduced, but not completely removed, in *maxcounts*. Plots for Jiang's data ("nucleus" libraries), depicted in Figure 2A, show no dependency of *maxcounts *on exon length. Conversely, for *maxcounts * in Griffith's and MAQC2 data sets a slight under-representation of exons shorter than 50 bp is still visible. We believe this behavior is explained by the difference in read length among the three data sets and the ability of TopHat to map them on splice junctions. Indeed, we observed that in MAQC2 and Griffith's data sets (36 bp reads) only 0.25-0.50% of aligned reads are mapped on splice junctions, as opposed to 2.5-11.5% of reads in Jiang's data set (75 bp reads). As a consequence, there is a decrease of counts over exons boundaries, which mainly affects short exons. In all the considered data sets, RPKM-normalized *totcounts *show a negative relationship with exon length due to an over-correction for length bias on short exons. On the opposite, full-quantile normalization completely removes exon length bias. Similarly, if applied to *maxcounts*, full-quantile normalization completely removes exon-length bias even on short exons (plots not shown).

We used the same approach to investigate GC-content effect, revealing a moderate bias due to GC-composition on almost all data sets (Figure 2B and Additional Files 2, 3, 4, 5, 6). As noted in previous studies, GC-content effect is not consistent across data sets [20,24,25,36]. Interestingly, the correction for exon length bias *via *full-quantile normalization also corrects for GC-content bias all the considered data sets.

In the following assessments, we always show raw *totcounts *and their RPKM- and full-quantile-normalized versions. Given the low length bias characterizing *maxcounts*, we consider their raw, not-normalized version.

### Bias due to highly expressed genes

**Figure 3:**
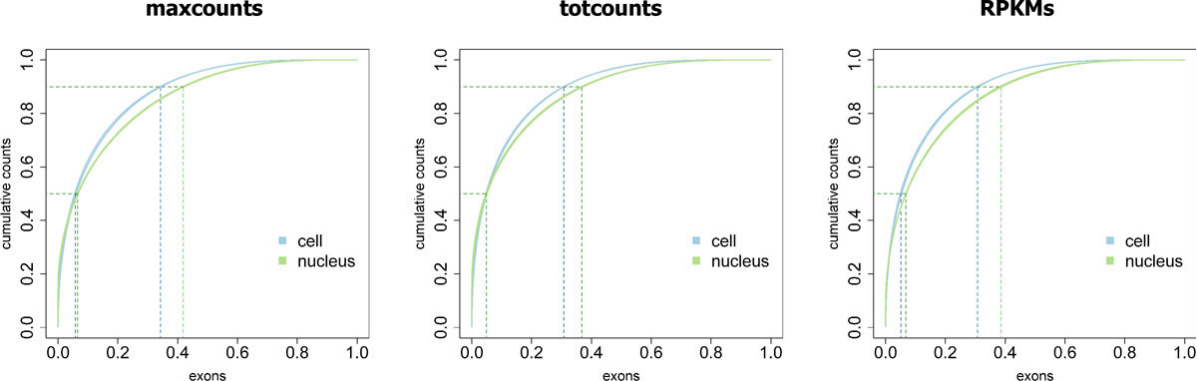
**Distribution of counts across exons**. Distribution of *maxcounts*, *totcounts *and RPKM-corrected *totcounts *(*RPKM*) across exons, in Jiang's data set. Plots represent cumulative counts/RPKMs (y-axis, percentage referred to total counts/RPKMs in a library) assigned to exons (x-axis, percentage referred to the number of exons with more than zero counts/RPKMs). Each curve represents one library and different colours identify different groups. Dashed lines represent 50% and 90% of total counts/RPKMs and are summarized in Table 1.

We assessed the distribution of counts to detect possible biases due to highly transcribed genes, which may affect detection power of differentially expressed exons [17,37]. As evident from Table 1, Figure 3 and Additional File 7, we confirm that most of the reads are generated by a small subset of highly expressed genes.

**Table 1:** Summary of count distribution across exons

			Exons (%)		
Data set	Group	counts (%)	maxcounts	totcounts	RPKM
**Jiang**	**Cell**	50	6	5	5
		90	34	31	31
	
	**Nucleus**	50	7	5	7
		90	42	37	39

**Griffith**	**MIP101**	50	9	4	8
		90	44	33	40
	
	**MIP5FU**	50	9	4	8
		90	45	33	40

**MAQC2**	**Brain**	50	6	3	5
		90	38	26	33
	
	**UHR**	50	5	3	4
		90	37	27	33

Summary of the distributions of *maxcounts*, *totcounts *and RPKM-corrected *totcounts *(*RPKM*) across exons in Jiang's, Griffith's and MAQC2 data sets. Table reports the percentage of exons accounting for 50% and 90% of total counts/RPKMs (average values across libraries belonging to the same condition).

In particular, Table 1 reports the percentage of exons accounting for 50% and 90% of total counts or RPKMs in a sample, highlighting that, less than 40% of exons contain more than 90% of all *totcounts *in a library. RPKM-normalized *totcounts *are more evenly distributed across exons, but the least biased distribution is that of *maxcounts*, with a marked improvement on the more biased data sets (see, for example, how this bias is reduced on Griffith's data).

### Quantification of spike-in RNAs

**Figure 4:**
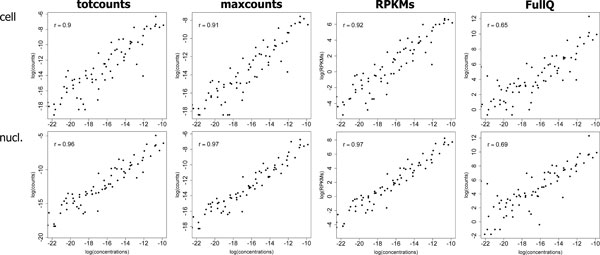
**Quantification of spike-in RNAs**. Counts/RPKMs obtained for spike-in RNAs from Jiang's data set, "cell" and "nucleus" groups, plotted against true concentrations (log-log scale). Counts/RPKMs are computed using *totcounts*, *maxcounts*, RPKM-corrected *totcounts *(*RPKM*) and *totcounts *corrected with within-lane full-quantile normalization over exon length (*FullQ*). "*r*" indicates Pearson's correlation (p-values always < 1e-11).

We estimated abundances of spike-in RNAs on Jiang's data, by averaging *totcounts *and *maxcounts *across all technical replicates within each group (Figure 4).

For all measures, plots show higher agreement with the gold-standard on Jiang's "nucleus" data, probably because of the higher number of replicates (six libraries) with respect to "cell" data (two libraries). All measures, with the exception of full-quantile-normalized *totcounts*, obtain high correlation with true concentrations, with RPKM-normalized *totcounts *and *maxcounts *having slightly better results than *totcounts*. Full-quantile normalization performed on *totcounts*, although eliminating length bias, possibly over-corrects data. Correlations with true concentrations of *maxcounts*, *totcounts *and RPKM-normalized *totcounts*, computed on all libraries of Jiang's data set, do not significantly differ (two-sided t-test, p-value > 0.05). On the contrary, full-quantile-normalized *totcounts *present the lowest correlation with spike-in RNAs concentrations (two-sided t-test, p-value < 1e-10). All methods do not depend on transcript abundances, except for full-quantile-normalized *totcounts*, which are less robust in estimating low-abundance transcripts (Additional File 8).

**Figure 5:**
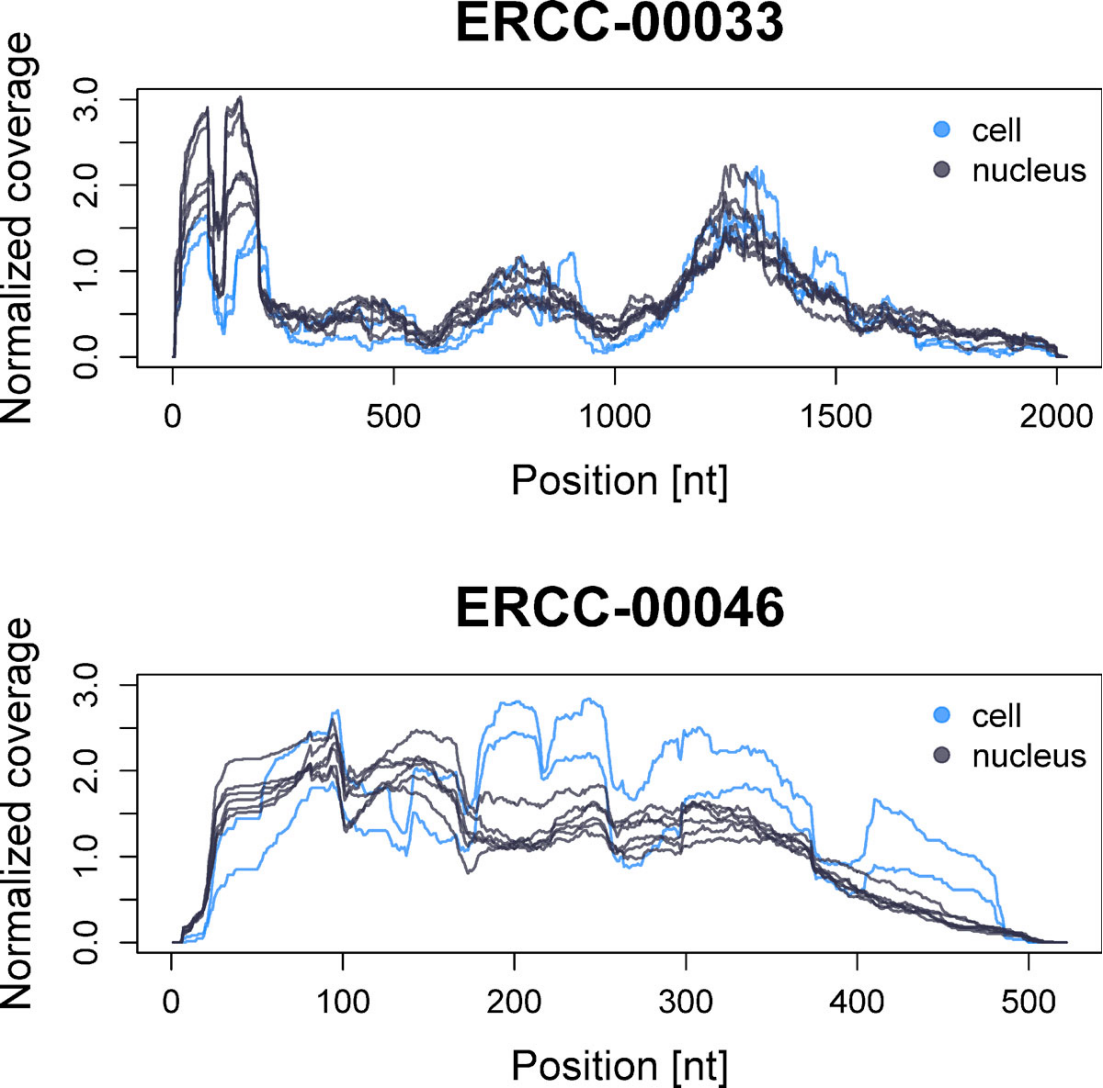
**Non uniform coverage of spike-in RNAs**. Read coverage (or "positional counts") along two spike-in RNAs, ERCC-00033 and ERCC-00046, in Jiang's libraries. "Cell" and "nucleus" replicates are indicated with blue and grey curves, respectively. Read coverage for each library is normalized to its sequencing depths.

Jiang's data set is particularly interesting because it allows the investigation of the non-uniformity of read coverage along spike-in RNAs, which was also reported in previous studies [28,31] (Figure 5). Changes in read coverage are not justified by alternative splicing since spike-in RNAs are single-isoform, and show reproducible patterns on the same transcript sequenced in different libraries and conditions. As previously noted by Li *et al. *[28], reads are not randomly sequenced from transcripts, but some positions present a larger "sequencing preference" and result in higher (positional) counts.

Figure 5 highlights differences in read coverage along two transcripts having very similar concentrations, ERCC-00033 (7.06-e-07 nmol/μl) and ERCC-00046 (7.08-e-07 nmol/μl), with the latter having a more uniform coverage. To have a measure of how much those patterns affect *maxcounts *and *totcounts *quantification (for which an overall comparison is given in the previous paragraph), we can compute the variation of *maxcounts*/*totcounts *estimates on these two transcripts as:

$$\text{Δ}=\frac{{\text{X}}_{33}-{\text{X}}_{46}}{{\text{X}}_{33}+{\text{X}}_{46}}\cdot 100$$

where X*_i _*are *totcounts *or *maxcounts*, averaged across libraries, for each transcript here considered. Ideally, Δ should be very small, to reflect the closeness of the true concentrations. Whereas *totcounts *produce a variation of 39%, *maxcounts *have a much smaller variation of 2%, overcoming read-coverage bias and providing very similar estimates for the transcripts here used as example. It is interesting to note that both transcripts show a reduced read coverage in correspondence to 3' end (Figure 5), a bias that is introduced during the reverse-transcription step performed with random hexamers (see "Background"). This bias is present in all transcripts of Jiang's data set (results not shown). *Maxcounts *approach is robust to 3' bias since it considers the bases with the highest read coverage along transcripts.

### Data variance

**Figure 6:**
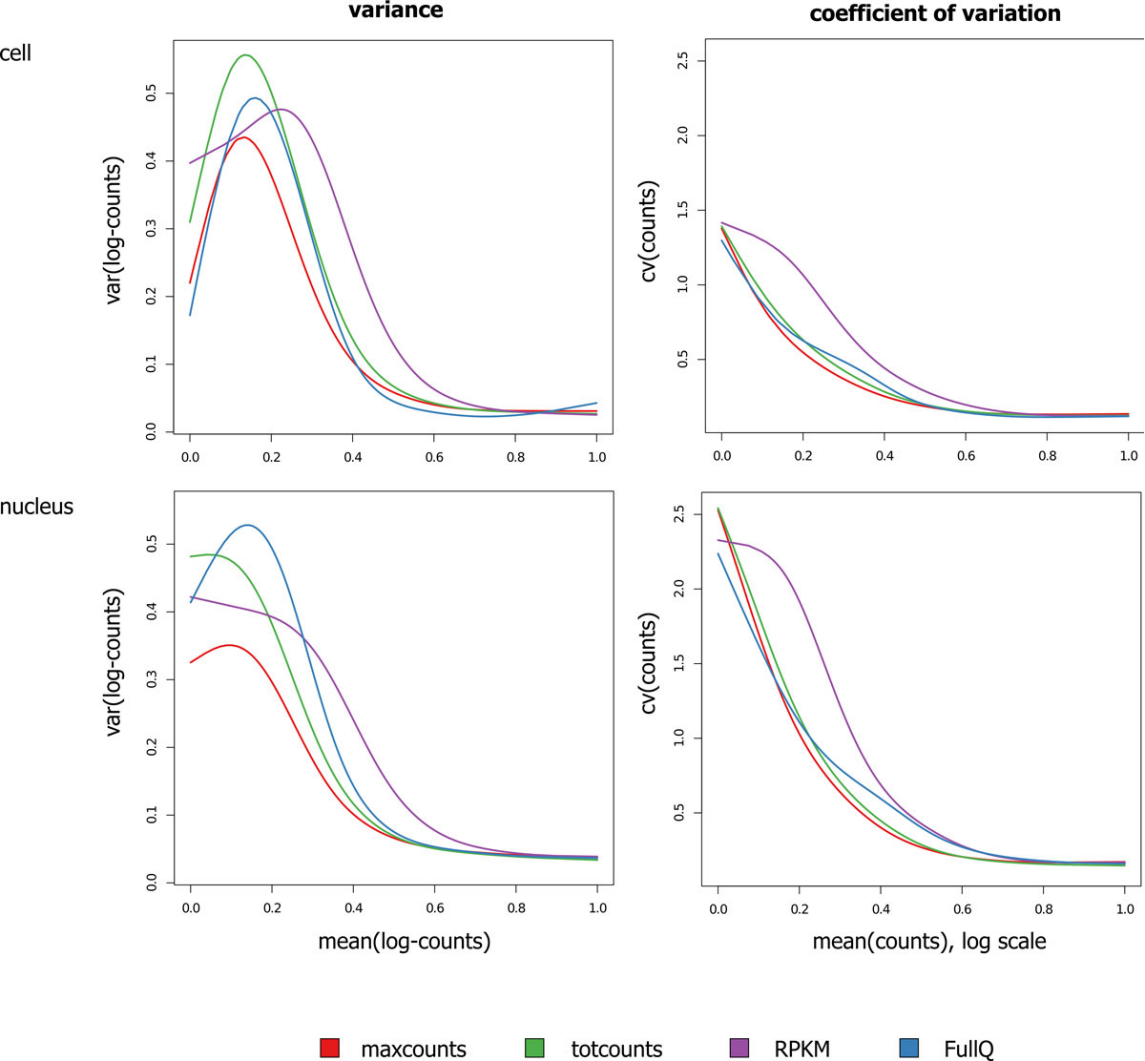
**Data variance and coefficient of variation**. Variance and coefficient of variation (CV) of Jiang's data: variance *vs*. mean of log-counts/RPKMs (left plots) and CV *vs*. log-mean of counts/RPKMs (right plots). Curves represent cubic-spline fits computed on variance/CV, averaged in bins of 5000 exons each. Since *maxcounts*, *totcounts*, and *totcounts *normalized with RPKM (*RPKM*) and within-lane full-quantile normalization over exon length (*FullQ*) approaches are compared, x-values are scaled to cover the range [0, 1] in order to make them comparable.

To easily compare variance of *totcounts *(and its normalized versions) versus *maxcounts*, at different expression intensities, we quantized the estimated average expression intensities in intervals of equal size and, for each interval, we calculated the average intensity and the average variance as explained in [38]. Finally we fitted data using a cubic spline (Figure 6 and Additional Files 9 and 10).

*Maxcounts *show the lowest variance at low and mean expressions, while *totcounts *present slightly lower variance at high expressions. In order to account for differences in the range of values, we also considered the coefficient of variation (CV, ratio between standard deviation and mean). *Totcounts *and *maxcounts *obtain comparable CV curves. *Totcounts *normalized with full-quantile are characterized by larger variance and CV with respect to both *maxcounts *and *totcounts*, while *totcounts *normalized with RPKM-normalized *totcounts *have the highest variance and CV.

### Robustness to alignment quality

An important criterion for the evaluation of reproducibility is the robustness of *totcounts *and *maxcounts *to variations in the quality of alignments. Results presented so far refer to a filtered set of alignments obtained using the analysis pipeline defined for this study, in which multireads and low-similarity alignments were discarded (see "Methods" for additional details). To investigate how this choice impacts on quantification, for each exon i in each library j, we measured the relative variation between counts X(i, j) obtained from the original set of alignments and from the filtered set, as follows:

$$relative\text{\_}variation=\frac{{\text{X}}_{orig}\left(\text{i},\text{j}\right)-{\text{X}}_{filt}\left(\text{i},\text{j}\right)}{{\text{X}}_{orig}\left(\text{i},\text{j}\right)+1}\cdot 100$$

where the expression at the denominator is used to avoid possible divisions by zero. Ideally, if a measure is stable to alignment filtering (that depends on the specific analysis pipeline defined by users), relative variation should be 0%. Here we consider raw *maxcounts *and *totcounts*, not subjected to any normalization, since we want to assess the direct impact that changes in alignment filtering have on count summarization.

**Figure 7:**
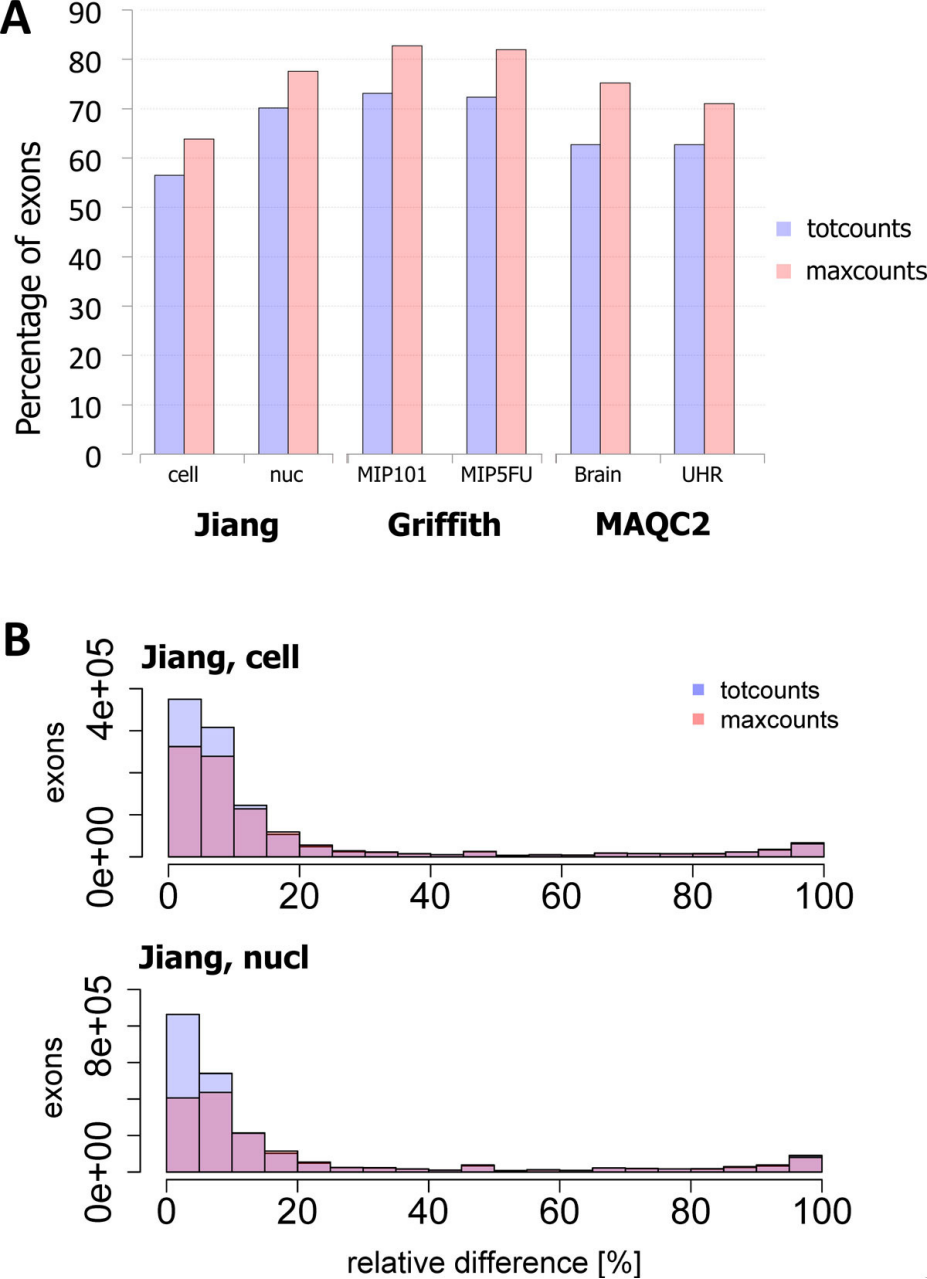
**Effect of alignment quality**. Relative variation of non-normalized *totcounts *(blue) and *maxcounts *(red) when low-similarity alignments and multireads are discarded: percentage of exons with null variation (**A**) and superimposed histograms of non-null variations affecting exons (**B**).

On all data sets, the fraction of exons for which *maxcounts *have 0% variation is always higher than that of *totcounts *(one-tailed t-test, p-value = 0.02). In particular, on Griffith's data, more than 80% of exons are not affected by alignment filtering (Figure 7A). In addition, histograms of relative variations show that only a small fraction of exons are affected by medium-high variation (Figure 7B and Additional File 11). For visualization purpose, exons with null variations are not represented by histograms, since they would result in a very high bar in correspondence of 0%, making it harder to assess variations greater than 0%.

Moreover, alignments filtering also impacts on the number of reads that can be used for quantification. Indeed, by applying this filter to any of the three data sets, about 30% of reads are discarded. Hence, the results reported above highlight that *maxcounts *are robust to variations in the number of considered reads.

## Conclusions

Thanks to the advent and progress of NGS technologies, RNA-seq has rapidly become the method of choice for measuring and comparing gene transcription levels. In this methodology, the expression of a coding unit, such as a gene, transcript or exon, is estimated by considering the total number of reads that can be aligned on its sequence (*totcounts*). Despite being widely adopted, this digital measure of expression is not free from biases, and efforts are underway by the scientific community to develop novel methods for data normalization and bias correction. Here we propose an alternative approach for computing RNA-seq counts: *maxcounts*. We exploit read coverage along an exon to compute *maxcounts *as the maximum of its positional counts, i.e. the number of reads covering each base along its sequence.

We characterized and compared *totcounts *and *maxcounts *considering the desired features of a measure of expression, irrespectively of downstream applications: no dependence on covariates, such as exon length and GC-content, no over-representation of highly transcribed exons, accurate and precise estimation of true expression levels, low variance and high reproducibility.

Overall, *totcounts *always need normalization for exon-length since they present a strong bias. On the contrary, exon-length bias in *maxcounts *is strongly reduced, so they do not necessarily require normalization. If exon-length bias is corrected through within-lane full-quantile normalization, further correction for GC-content is not needed neither for *totcounts *nor for *maxcounts*. Moreover, with *maxcounts *the over-representation of highly expressed exons is reduced with respect to *totcounts*. When focusing on accuracy and precision of measurements, *maxcounts *together with RPKM-corrected *totcounts *most accurately reproduce real data, whereas *maxcounts *together with *totcounts *normalized with the full-quantile approach show the lowest variance. Finally, although the quality of alignments has a great impact on both methods, *maxcounts *approach outperforms *totcounts *in terms of robustness to variations in alignment filtering.

Consequently, we believe that *maxcounts *approach represents a valuable alternative to *totcounts *for measuring exon expression from RNA-seq data, since it has comparable or higher performance on all the evaluation criteria.

Although several improvements have been made to understand and correct for possible biases in the RNA-seq experimental protocol, read coverage along transcripts still shows sequence-specific variability and under-representation of specific regions. *Maxcounts *approach can overcome biases due to the non-uniformity of read coverage, selecting the best-represented transcript regions. Nevertheless, RNA-seq is a methodology still under active development, which will experience a fast improvement of experimental protocols and evolution of data characteristics. We made available the code for calculating *maxcounts *(see "Methods"), thus enabling its benchmarking on different data sets.

A possible limitation of the current implementation is represented by the use of exons, since the final user might be interested in a having gene or transcript counts. Future work will focus on the definition of transcription models that can be used to combine exon *maxcounts *into an accurate measure of gene or transcript expression. Finally, an important issue to be addressed in the near future is the impact of *maxcounts *on differential expression analysis. At the moment, a complete assessment is difficult because of the lack of good benchmarks: microarrays and quantitative PCR can be used to measure *maxcounts *precision, but might not capture the complete picture of gene expression since they present a lower sensitivity with respect to RNA-seq. For these reasons, we are currently generating an *ad hoc *data set to assess both differential expression at exon and transcript level and to focus on expression of alternative splicing variants.

## List of abbreviations

cDNA: complementary DNA; NGS: Next-Generation Sequencing; RNA-seq: RNA sequencing; RPKM: Reads Per Kilobase of exon Model per million mapped reads; TMM: Trimmed Mean of M-values.

## Competing interests

The authors declare that they have no competing interests.

## Authors' contributions

FF performed bioinformatics and statistics analyses, implemented *maxcounts *code and drafted the manuscript. EL, LBI and PF contributed to the bioinformatic analysis and helped to draft the manuscript. PM contributed to the bioinformatic analysis and to the implementation of *maxcounts *code. ST and LBA contributed to the discussions and study design, and helped to draft the manuscript. BDC contributed to the discussions and to the study design, coordinated the study and helped to draft the manuscript. All authors read and approved the final manuscript.

## Supplementary Material

Additional file 1 **Supplementary materials and methods**. Click here for file

Additional file 2 **Exon length bias and GC-content effect (Jiang, "cell")**. Smoothed scatter-plots showing the relationship between log-counts/RPKMs and exon length (log scale, **A**) or GC-content (**B**), in Jiang's data ("cell" libraries). The orange curve represents a cubic-spline fit computed on log-counts, averaged in bins of 5000 exons each (black crosses between vertical lines, indicating bin bounds). Counts or RPKMs are computed using *totcounts*, *maxcounts*, RPKM-corrected *totcounts *(*RPKM*) and *totcounts *corrected with within-lane full-quantile normalization over exon length (*FullQ*), and averaged across libraries. Click here for file

Additional file 3 **Exon length bias and GC-content effect (Griffith, "MIP5FU")**. Smoothed scatter-plots showing the relationship between log-counts/RPKMs and exon length (log scale, **A**) or GC-content (**B**), in Griffith's data ("MIP5FU" libraries). The orange curve represents a cubic-spline fit computed on log-counts, averaged in bins of 5000 exons each (black crosses between vertical lines, indicating bin bounds). Counts or RPKMs are computed using *totcounts*, *maxcounts*, RPKM-corrected *totcounts *(*RPKM*) and *totcounts *corrected with within-lane full-quantile normalization over exon length (*FullQ*), and averaged across libraries. Click here for file

Additional file 4 **Exon length bias and GC-content effect (Griffith, "MIP101")**. Smoothed scatter-plots showing the relationship between log-counts/RPKMs and exon length (log scale, **A**) or GC-content (**B**), in Griffith's data ("MIP101" libraries). The orange curve represents a cubic-spline fit computed on the average log-counts in bins of 5000 exons each (black crosses between vertical lines, indicating bin bounds). Counts or RPKMs are computed using *totcounts*, *maxcounts*, RPKM-corrected *totcounts *(*RPKM*) and *totcounts *corrected with within-lane full-quantile normalization over exon length (*FullQ*), and averaged across libraries. Click here for file

Additional file 5 **Exon length bias and GC-content effect (MAQC2, "Brain")**. Smoothed scatter-plots showing the relationship between log-counts/RPKMs and exon length (log scale, **A**) or GC-content (**B**), in MAQC2 data ("Brain" libraries). The orange curve represents a cubic-spline fit computed on log-counts, averaged in bins of 5000 exons each (black crosses between vertical lines, indicating bin bounds). Counts or RPKMs are computed using *totcounts*, *maxcounts*, RPKM-corrected *totcounts *(*RPKM*) and *totcounts *corrected with within-lane full-quantile normalization over exon length (*FullQ*), and averaged across libraries. Click here for file

Additional file 6 **Exon length bias and GC-content effect (MAQC2, "UHR")**. Smoothed scatter-plots showing the relationship between log-counts/RPKMs and exon length (log scale, **A**) or GC-content (**B**), in MAQC2 data ("UHR" libraries). The orange curve represents a cubic-spline fit computed on log-counts, averaged in bins of 5000 exons each (black crosses between vertical lines, indicating bin bounds). Counts or RPKMs are computed using *totcounts*, *maxcounts*, RPKM-corrected *totcounts *(*RPKM*) and *totcounts *corrected with within-lane full-quantile normalization over exon length (*FullQ*), and averaged across libraries. Click here for file

Additional file 7 **Distribution of counts across exons**. Distribution of *maxcounts*, *totcounts *and RPKM-corrected *totcounts *(*RPKM*) across exons, in Griffith's and MAQC2 data sets. Plots represent cumulative counts/RPKMs (y-axis, percentage referred to total counts/RPKMs in a library) assigned to exons (x-axis, percentage referred to the number of exons with more than zero counts/RPKMs). Each curve represents one library and different colours identify different groups. Dashed lines represent 50% and 90% of total counts/RPKMs and are summarized in Table 1. Click here for file

Additional file 8 **Quantification of spike-in RNAs: residues**. Quantification of spike-in RNAs concentrations, in all libraries of Jiang's data set, with *totcounts*, *maxcounts*, RPKM-corrected *totcounts *(*RPKM*) and *totcounts *corrected with within-lane full-quantile normalization over exon length (*FullQ*). Plots show the residues of the linear regression of counts/RPKMs over true concentrations (log-log scale), plotted against true concentrations in log scale. Click here for file

Additional file 9 **Data variance and coefficient of variation (MAQC2)**. Variance and coefficient of variation (CV) of MAQC2 data: variance *vs*. mean of log-counts/RPKMs (left plots) and CV *vs*. log-mean of counts/RPKMs (right plots). Curves represent cubic-spline fits computed on variances/CVs, averaged in bins of 5000 exons each. Since *maxcounts*, *totcounts*, and *totcounts *normalized with RPKM (*RPKM*) and within-lane full-quantile normalization over exon length (*FullQ*) approaches are compared, x-values are scaled to cover the range [0, 1] in order to make them comparable. Click here for file

Additional file 10 **Data variance and coefficient of variation (Griffith)**. Variance and coefficient of variation (CV) of Griffith's data: variance *vs*. mean of log-counts/RPKMs (left plots) and CV *vs*. log-mean of counts/RPKMs (right plots). Curves represent cubic-spline fits computed on variances/CVs, averaged in bins of 5000 exons each. Since *maxcounts*, *totcounts*, and *totcounts *normalized with RPKM (*RPKM*) and full-quantile (*FullQ*) approaches are compared, x-values are scaled to cover the range [0, 1] in order to make them comparable. Click here for file

Additional file 11 **Effect of alignment quality**. Superimposed histograms of relative variation of non-normalized *totcounts *(blue) and *maxcounts *(red) when low-similarity alignments and multireads are discarded (only null-variations are reported) for MAQC2 (**A**) and Griffith's data (**B**). Click here for file

## Declarations

The publication costs for this article were funded by PRAT 2010 CPDA101217 ("Models of RNA sequencing data variability for quantitative transcriptomics").

This article has been published as part of *BMC Bioinformatics *Volume 15 Supplement 1, 2014: Integrated Bio-Search: Selected Works from the 12th International Workshop on Network Tools and Applications in Biology (NETTAB 2012). The full contents of the supplement are available online at http://www.biomedcentral.com/bmcbioinformatics/supplements/15/S1.
