# Supplementary material for: Reducing bias in RNA sequencing data: a novel approach to compute counts
Source: BMC Bioinformatics. 2014 Jan 10;15(Suppl 1):S7. doi: 10.1186/1471-2105-15-S1-S7 (PMC4016203; doi:10.1186/1471-2105-15-S1-S7)
Supplement: Additional file 1 — Supplementary materials and methods. [file 1471-2105-15-S1-S7-S1.pdf]

Reducing bias in RNA sequencing data: a novel approach to compute counts

Francesca Finotello[1], Enrico Lavezzo[2], Luca Bianco[3], Luisa Barzon[2], Paolo Mazzon[1], Paolo Fontana[3], Stefano Toppo[2], Barbara Di Camillo[1§]

[1]Department of Information Engineering, University of Padova, Padova, 35131, Italy

[2]Department of Molecular Medicine, University of Padova, Padova, 35131, Italy

[3]Research and Innovation Centre, Edmund Mach Foundation, Trento, 38010, Italy

[§]Corresponding author

Email addresses:

FF: francesca.finotello@dei.unipd.it

EL: enrico.lavezzo@unipd.it

LBI: luca.bianco@fmach.it

LBA: luisa.barzon@unipd.it

PM: mazzon@dei.unipd.it

PF: paolo.fontana@fmach.it

ST: stefano.toppo@unipd.it

BDC: dicamill@dei.unipd.it

Supplementary materials and methods

Data and annotation

The MAQC2 data set [1], generated for the MicroArray quality control (MAQC) project, contains expression data from multiple platforms. Here we consider RNA-seq data, which are available from the NCBI Sequence Read Archive [2] [SRA: SRA010153]. This data set consists in 36-bp single-end reads obtained by sequencing with the Solexa 1G Genome Analyzer two different biological samples: (i) Ambion's Human Brain Reference RNA ("Brain"), a standard pooled from multiple donors and several brain regions; (ii) Stratagene's Universal Human Reference RNA ("UHR"), a mixture of total RNA extracted from ten different human cell lines. "Brain" and "UHR" samples were subjected to the same library preparation protocol and sequenced in seven lanes of two flow-cells (technical replicates).

In Griffith et al. [3], two fluorouracil (5-FU)-resistant ("MIP5FU") and (5-FU)-sensitive ("MIP101") human colorectal cancer cell lines were sequenced on 16 and 23 lanes of Illumina Genome Analyzer, respectively. Here we consider eight "MIP101" and eight "MIP5FU" libraries of 36bp paired-end reads; FASTQ files were kindly provided by Dr. Malachi Griffith.

In Jiang et al. [4] different Human ENCODE libraries were mixed with the Phase IV test set of ERCC RNA standards. The human samples mixed with ERCC RNAs were sequenced on the Illumina GAIIx platform to generate paired-end reads (2x76bp length). Here we consider RNA-seq data obtained from a K-562 cell line, extracted from nucleus or whole-cell. In particular, we consider two libraries from the "cell" group [SRA: SRR307930, SRR307931] and six libraries from the "nucleus" group [SRA: SRR317042, SRR317043].

We consider the GRCh37.p10 human reference genome and the Gencode human exon annotation (version 12), for mapping, computing counts and gathering information about GC-content.

Read mapping and alignment filtering

For read mapping, we used TopHat [5], version v2.0.6, using default parameter settings. When mapping single-end reads, we used the `-j` option to provide TopHat with the file of junctions obtained after paired-ends mapping. The expected distance between paired-end reads was estimated using PASS [6] as in [7].

Multireads and reads whose similarity with the reference was lower than 97% were discarded using SAMsieve, a java alignment-filtering program developed in house based on SAMtools API. It can filter alignments stored in SAM or BAM files based on several criteria (that can be easily combined) such as number of alignments reported, alignment quality, chromosome, number of mismatches, read coverage, percentage of identity, etc. In this work, to discard multireads and reads whose similarity with the reference was lower than 97%, the following options were used: "`reportedAligns4read<=,1`" and "`identity>=0.97`". SAMsieve is available upon request.

Computation of counts and normalization

Totcounts were computed using the `coverageBed` utility of bedtools [8], option `–counts`. We implemented a new functionality for bedtools' routine `coverageBed` that allows the computation of *maxcounts*, through two new options: `-max` and `–maxm` (the latter allows the user to select the number of exon positions, among those with the highest read coverages, to be used in the computation of *maxcounts*). This functionality is available as a patch for bedtools that can be downloaded from http://www.dei.unipd.it/~finotell/maxcounts/. The code is distributed along with

`formatCounts.sh`, a bash script for creating a matrix of counts starting from multiple files, from different libraries, generated with coverageBed (options: `-counts`, `-max`, `-maxm`).

Positional counts along spike-in RNAs were computed using `coverageBed`, option `-d`.

Differences in library sizes of *totcounts* and *maxcounts* replicates were corrected through Trimmed Mean of M-values (TMM) normalization [9] using edgeR [10]. We also tested the "relative log expression" (RLE) normalization [11], also implemented in edgeR, obtaining almost identical results (results not shown).

As described in the main text, within-lane full-quantile normalization of *totcounts* on exon length was performed using EDASeq [21]. In order to correct for differences in library sizes, this normalization was used together with between-lane full-quantile normalization, also implemented in EDASeq.

References

1. Bullard JH, Purdom E, Hansen KD, Dudoit S: **Evaluation of statistical methods for normalization and differential expression in mRNA-Seq experiments.** *BMC Bioinformatics* 2010, **11**:94.

2. [http://www.ncbi.nlm.nih.gov/sra/]

3. Griffith M, Griffith OL, Mwenifumbo J, Goya R, Morrissy AS, Morin RD, Corbett R, Tang MJ, Hou YC, Pugh TJ: **Alternative expression analysis by RNA sequencing.** *Nat Methods* 2010, **7**(10):843-847.

4. Jiang L, Schlesinger F, Davis CA, Zhang Y, Li R, Salit M, Gingeras TR, Oliver B: **Synthetic spike-in standards for RNA-seq experiments.** *Genome Res* 2011, **21**(9):1543-1551.

5. Trapnell C, Pachter L, Salzberg SL: **TopHat: discovering splice junctions with RNA-Seq.** *Bioinformatics* 2009, **25**(9):1105-1111.

6. Campagna D, Albiero A, Bilardi A, Caniato E, Forcato C, Manavski S, Vitulo N, Valle G: **PASS: a program to align short sequences.** *Bioinformatics* 2009, **25**(7):967-968.

7. Finotello F, Lavezzo E, Fontana P, Peruzzo D, Albiero A, Barzon L, Falda M, Di Camillo B, Toppo S: **Comparative analysis of algorithms for whole-genome assembly of pyrosequencing data.** *Brief Bioinform* 2012, **13**(3):269-280.

8. Quinlan AR, Hall IM: **BEDTools: a flexible suite of utilities for comparing genomic features.** *Bioinformatics* 2010, **26**(6):841-842.

9. Robinson MD, Oshlack A: **A scaling normalization method for differential expression analysis of RNA-seq data.** *Genome Biol* 2010, **11**(3):R25.

10. Robinson MD, McCarthy DJ, Smyth GK: **edgeR: a Bioconductor package for differential expression analysis of digital gene expression data.** *Bioinformatics* 2010, **26**(1):139-140.

11. Anders S, Huber W: **Differential expression analysis for sequence count data.** *Genome Biol* 2010, **11**(10):R106.
