# Supplementary figures and images for: Reducing bias in RNA sequencing data: a novel approach to compute counts
Source: BMC Bioinformatics. 2014 Jan 10;15(Suppl 1):S7. doi: 10.1186/1471-2105-15-S1-S7 (PMC4016203; doi:10.1186/1471-2105-15-S1-S7)

**A****totcounts**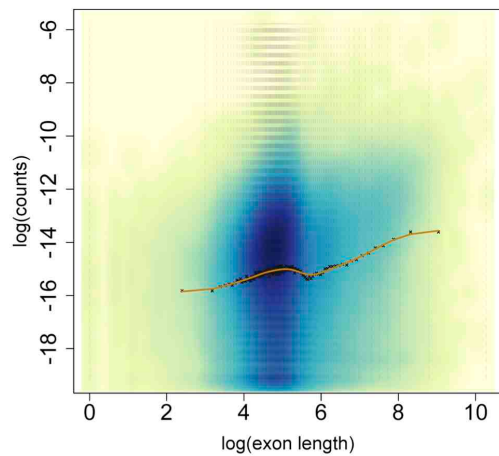**maxcounts**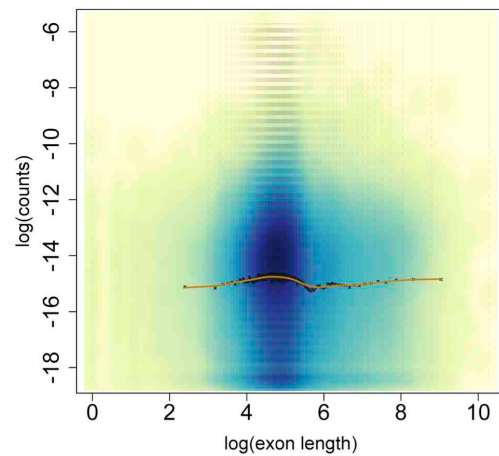**RPKM**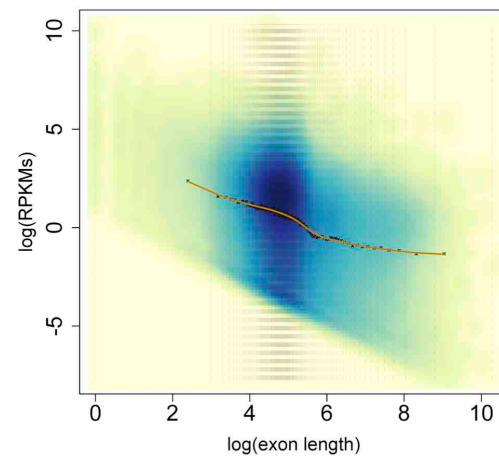**FullQ**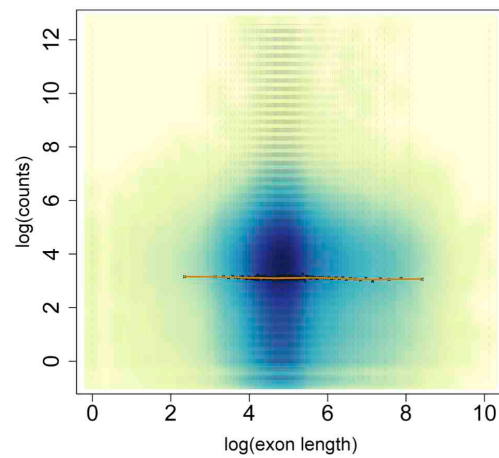**B****totcounts**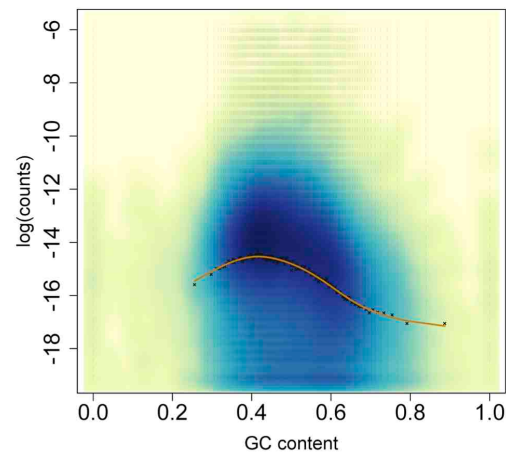**maxcounts**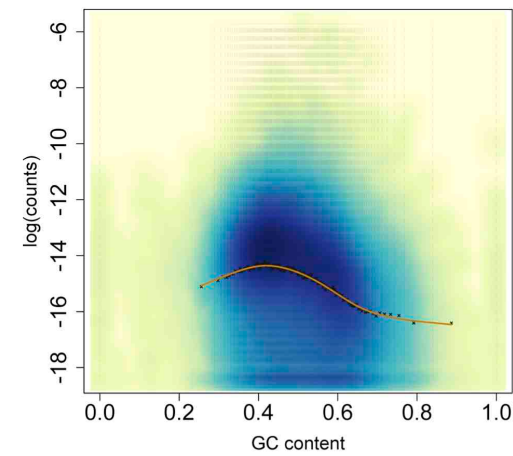**RPKM**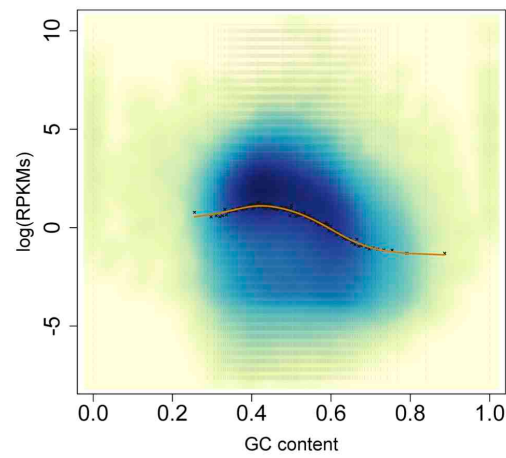**FullQ**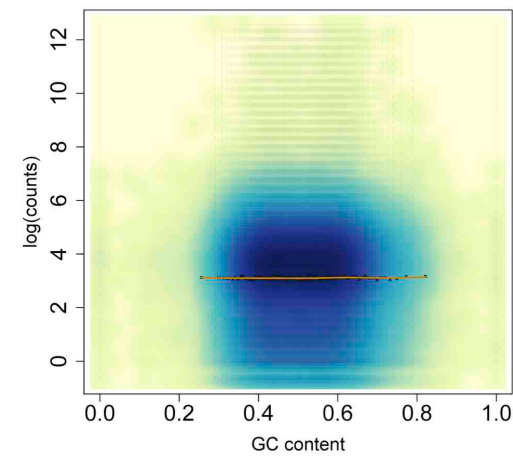

Supplement: Additional file 2 — Exon length bias and GC-content effect (Jiang, "cell"). Smoothed scatter-plots showing the relationship between log-counts/RPKMs and exon length (log scale, A) or GC-content (B), in Jiang's data ("cell" libraries). The orange curve represents a cubic-spline fit computed on log-counts, averaged in bins of 5000 exons each (black crosses between vertical lines, indicating bin bounds). Counts or RPKMs are computed using totcounts, maxcounts, RPKM-corrected totcounts (RPKM) and totcounts corrected with within-lane full-quantile normalization over exon length (FullQ), and averaged across libraries. [file 1471-2105-15-S1-S7-S2.pdf]

**A****totcounts**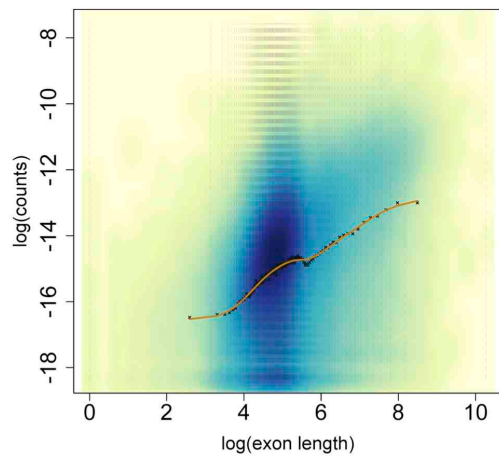**maxcounts**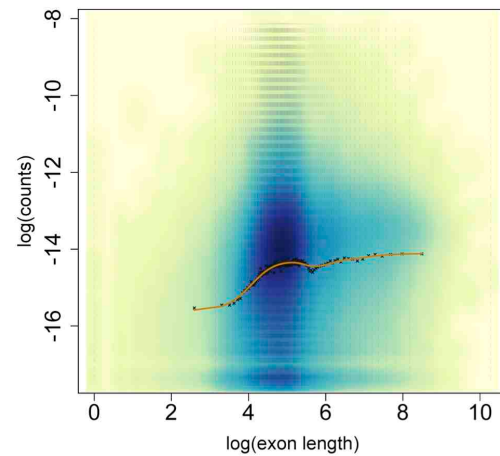**RPKM**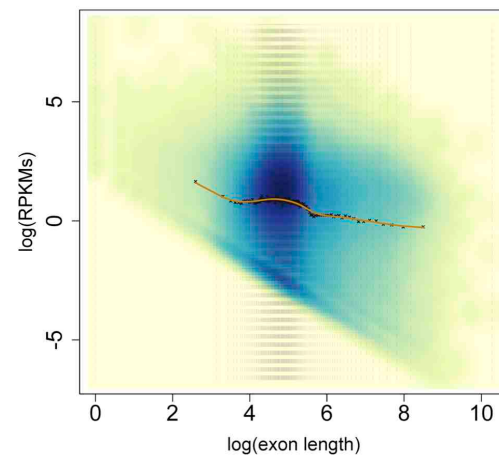**FullQ**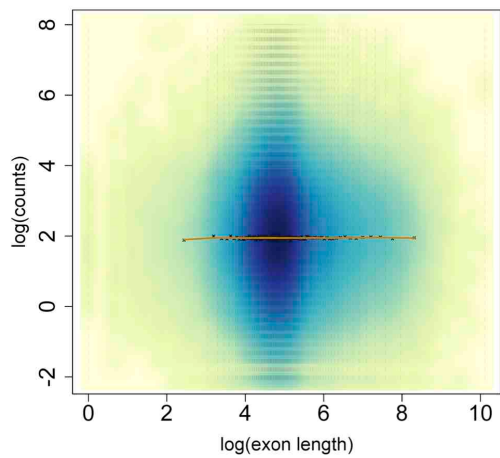**B****totcounts**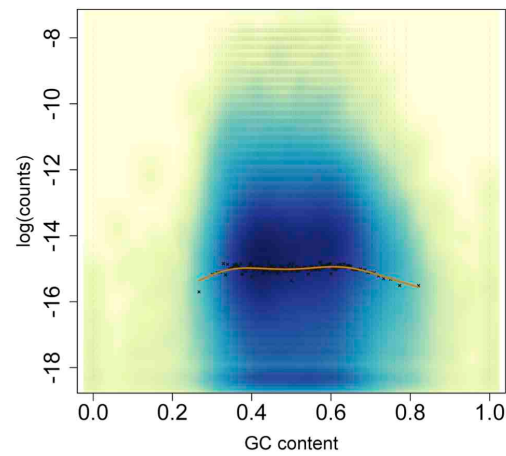**maxcounts**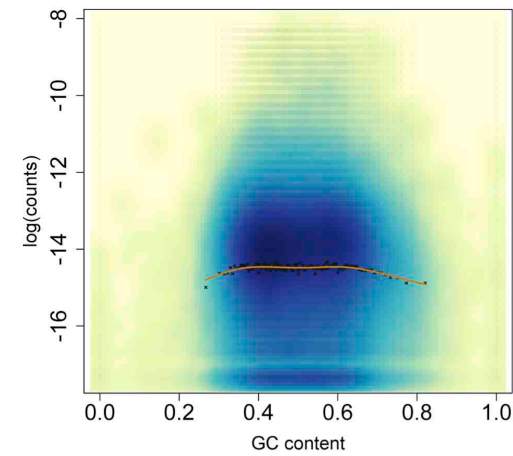**RPKM**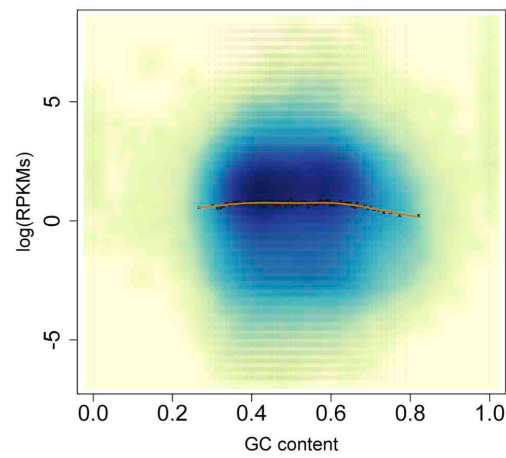**FullQ**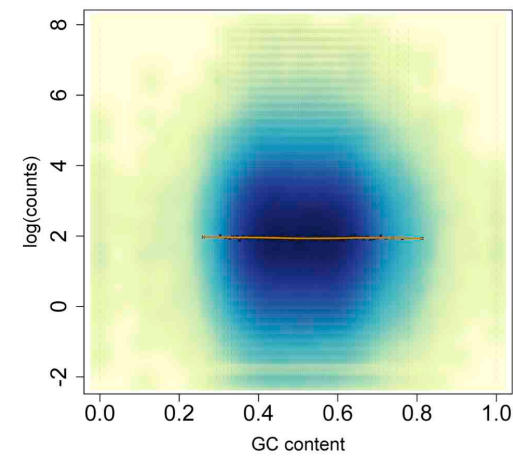

Supplement: Additional file 3 — Exon length bias and GC-content effect (Griffith, "MIP5FU"). Smoothed scatter-plots showing the relationship between log-counts/RPKMs and exon length (log scale, A) or GC-content (B), in Griffith's data ("MIP5FU" libraries). The orange curve represents a cubic-spline fit computed on log-counts, averaged in bins of 5000 exons each (black crosses between vertical lines, indicating bin bounds). Counts or RPKMs are computed using totcounts, maxcounts, RPKM-corrected totcounts (RPKM) and totcounts corrected with within-lane full-quantile normalization over exon length (FullQ), and averaged across libraries. [file 1471-2105-15-S1-S7-S3.pdf]

**A****totcounts**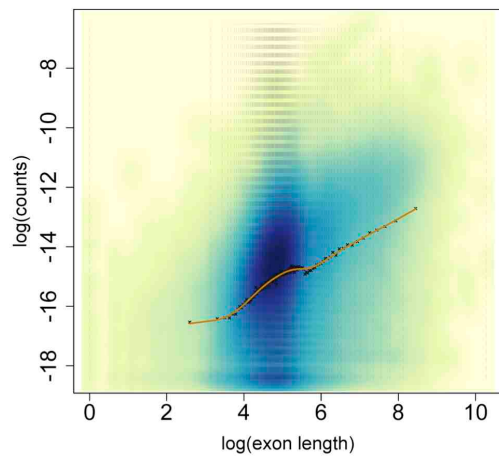**maxcounts**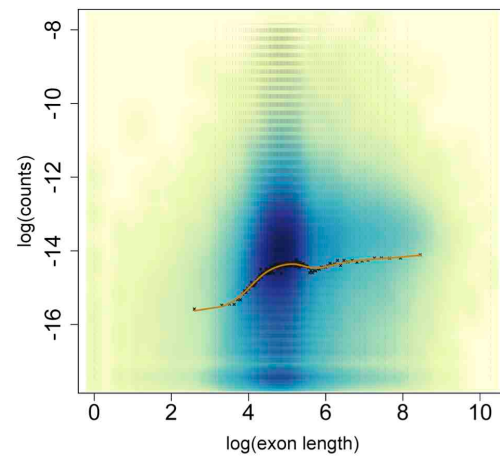**RPKM**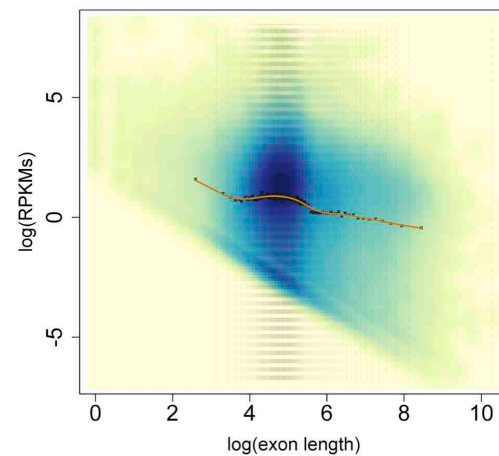**FullQ**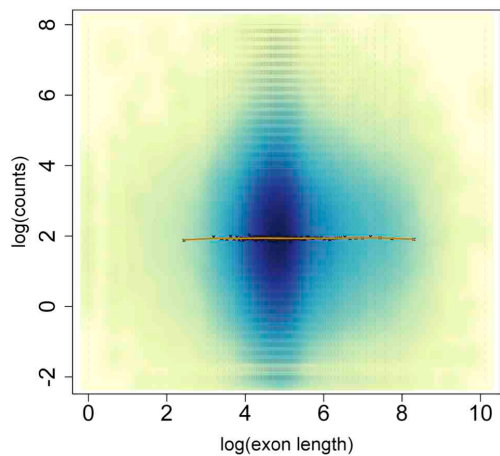**B****totcounts**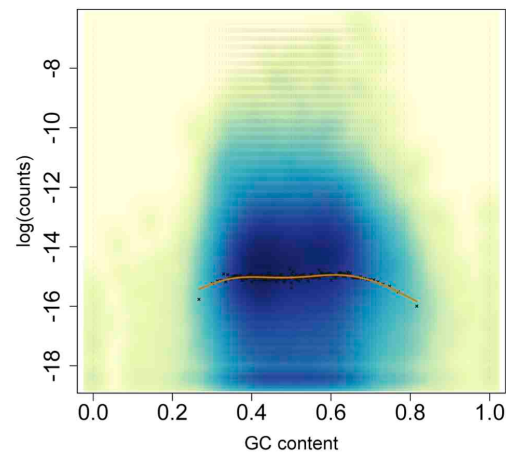**maxcounts**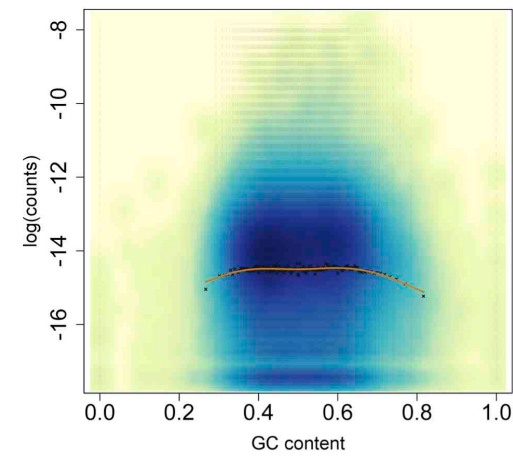**RPKM**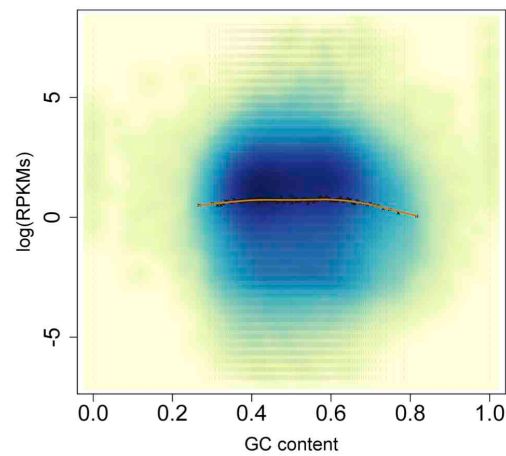**FullQ**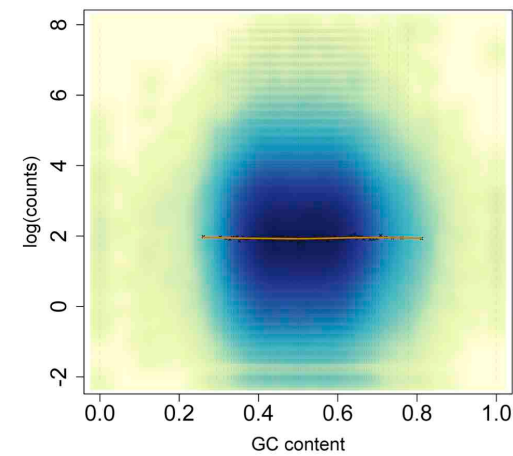

Supplement: Additional file 4 — Exon length bias and GC-content effect (Griffith, "MIP101"). Smoothed scatter-plots showing the relationship between log-counts/RPKMs and exon length (log scale, A) or GC-content (B), in Griffith's data ("MIP101" libraries). The orange curve represents a cubic-spline fit computed on the average log-counts in bins of 5000 exons each (black crosses between vertical lines, indicating bin bounds). Counts or RPKMs are computed using totcounts, maxcounts, RPKM-corrected totcounts (RPKM) and totcounts corrected with within-lane full-quantile normalization over exon length (FullQ), and averaged across libraries. [file 1471-2105-15-S1-S7-S4.pdf]

**A****totcounts**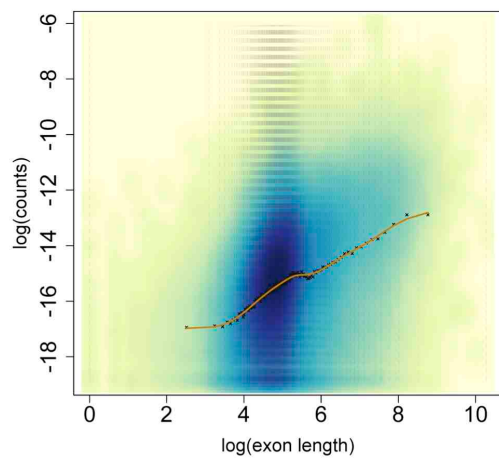**maxcounts**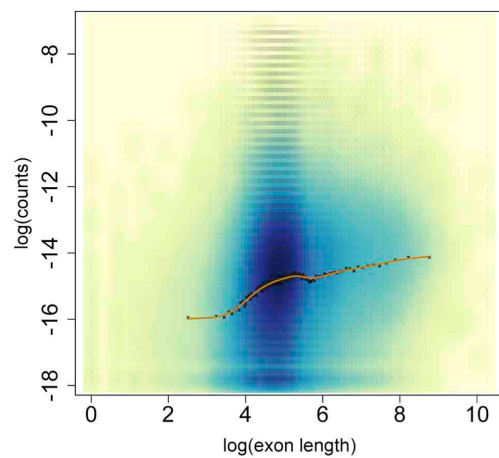**RPKM**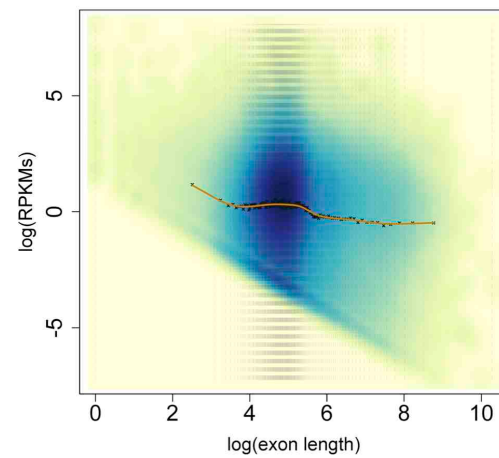**FullQ**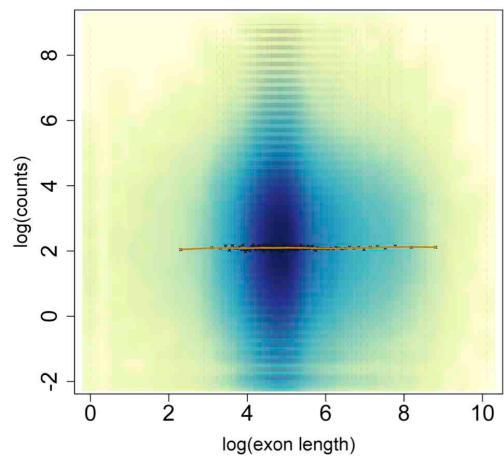**B****totcounts**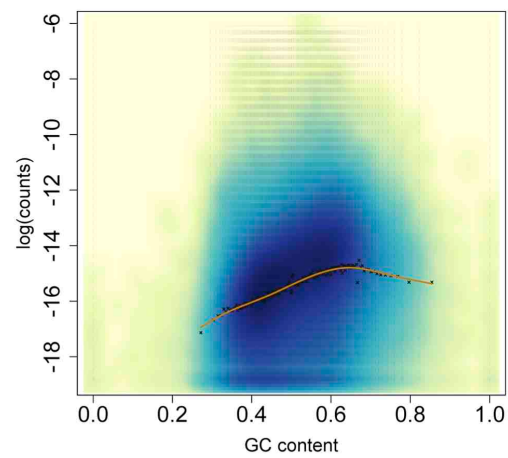**maxcounts**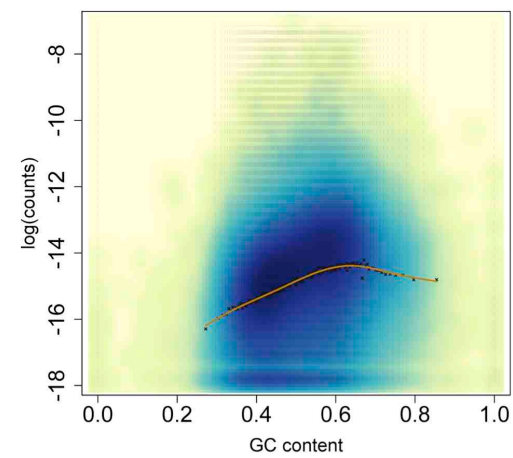**RPKM**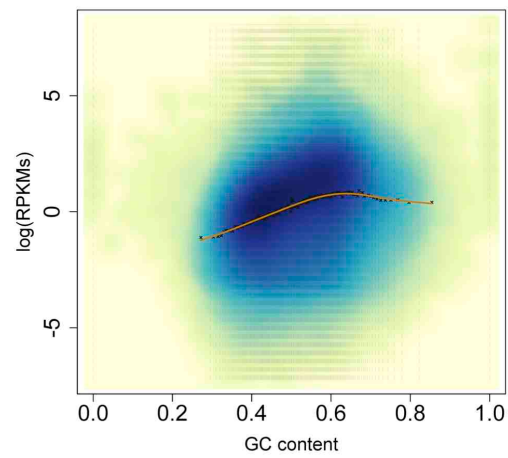**FullQ**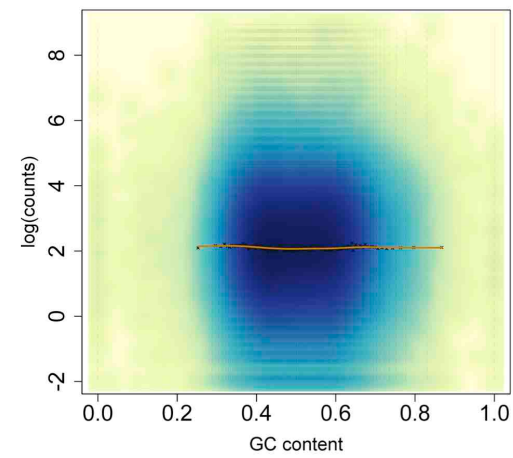

Supplement: Additional file 5 — Exon length bias and GC-content effect (MAQC2, "Brain"). Smoothed scatter-plots showing the relationship between log-counts/RPKMs and exon length (log scale, A) or GC-content (B), in MAQC2 data ("Brain" libraries). The orange curve represents a cubic-spline fit computed on log-counts, averaged in bins of 5000 exons each (black crosses between vertical lines, indicating bin bounds). Counts or RPKMs are computed using totcounts, maxcounts, RPKM-corrected totcounts (RPKM) and totcounts corrected with within-lane full-quantile normalization over exon length (FullQ), and averaged across libraries. [file 1471-2105-15-S1-S7-S5.pdf]

**A****totcounts**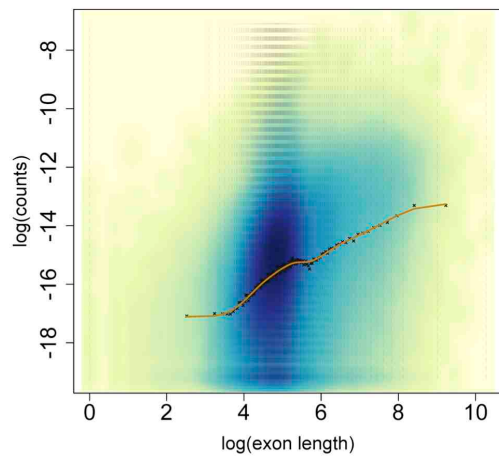**maxcounts**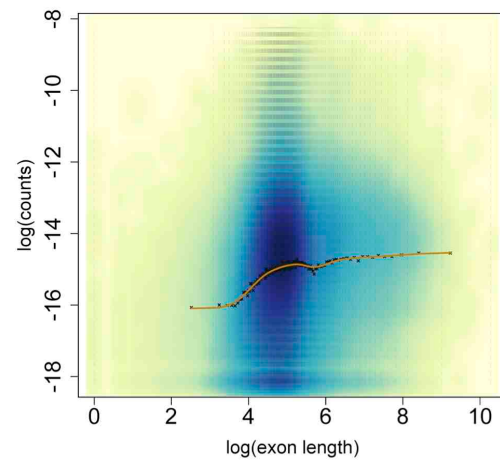**RPKM**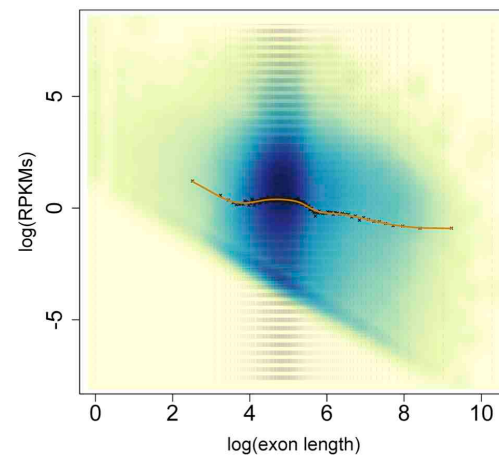**FullQ**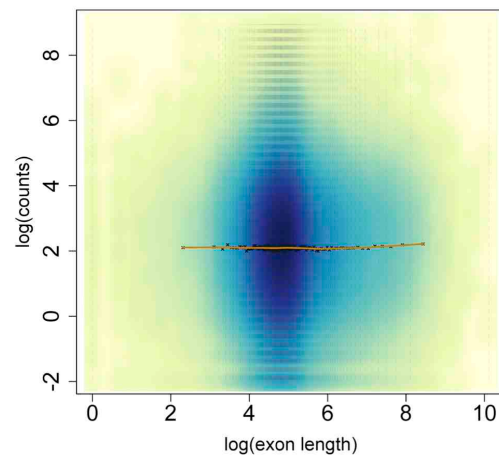**B****totcounts**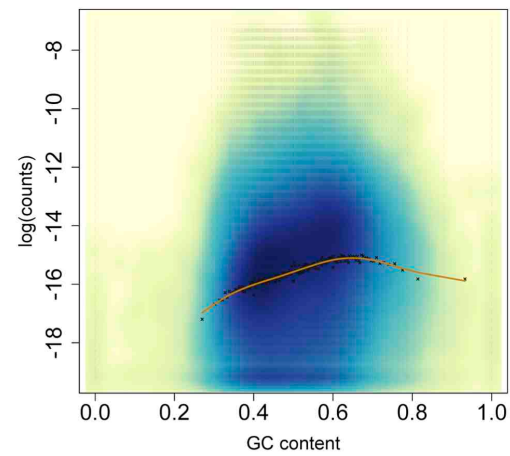**maxcounts**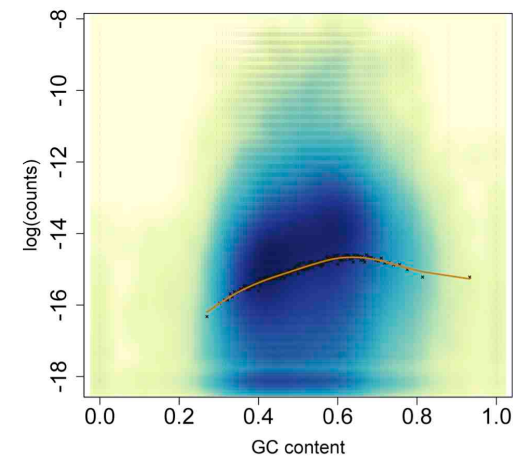**RPKM**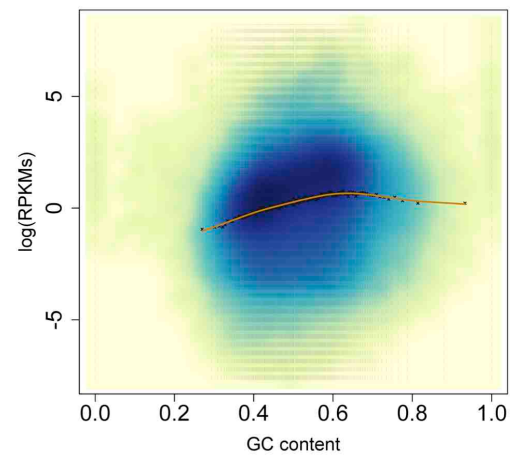**FullQ**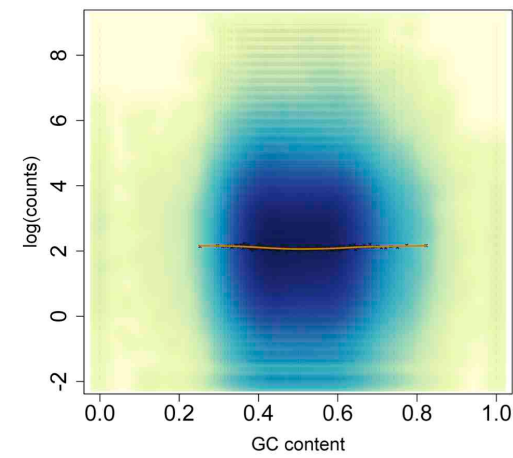

Supplement: Additional file 6 — Exon length bias and GC-content effect (MAQC2, "UHR"). Smoothed scatter-plots showing the relationship between log-counts/RPKMs and exon length (log scale, A) or GC-content (B), in MAQC2 data ("UHR" libraries). The orange curve represents a cubic-spline fit computed on log-counts, averaged in bins of 5000 exons each (black crosses between vertical lines, indicating bin bounds). Counts or RPKMs are computed using totcounts, maxcounts, RPKM-corrected totcounts (RPKM) and totcounts corrected with within-lane full-quantile normalization over exon length (FullQ), and averaged across libraries. [file 1471-2105-15-S1-S7-S6.pdf]

## Dataset

Griffith

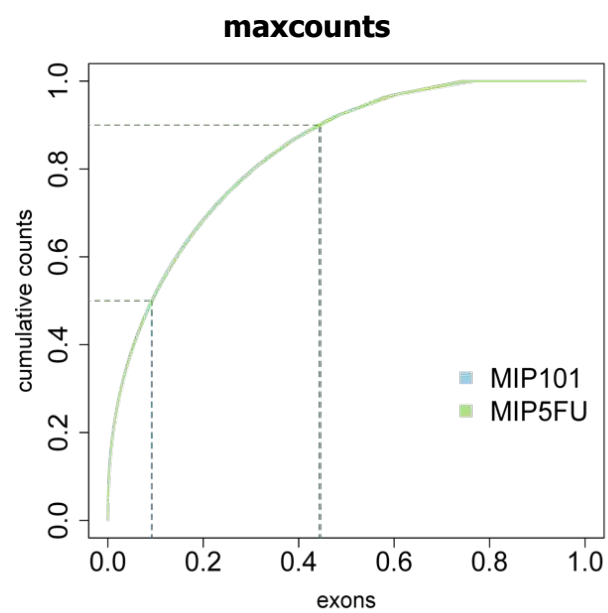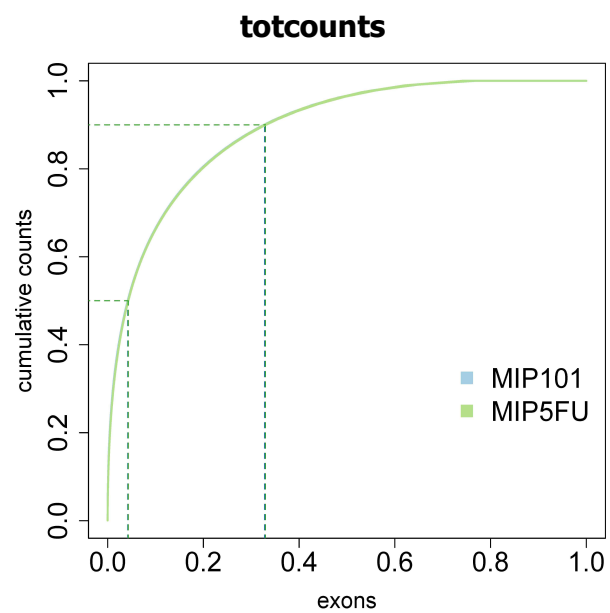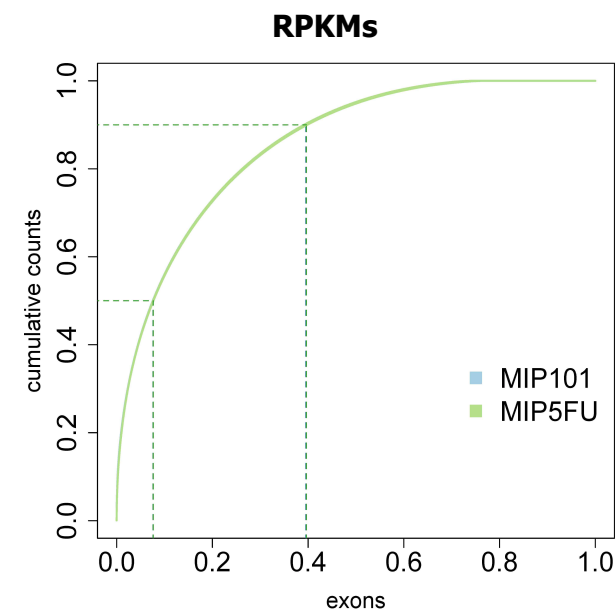

MAQC2

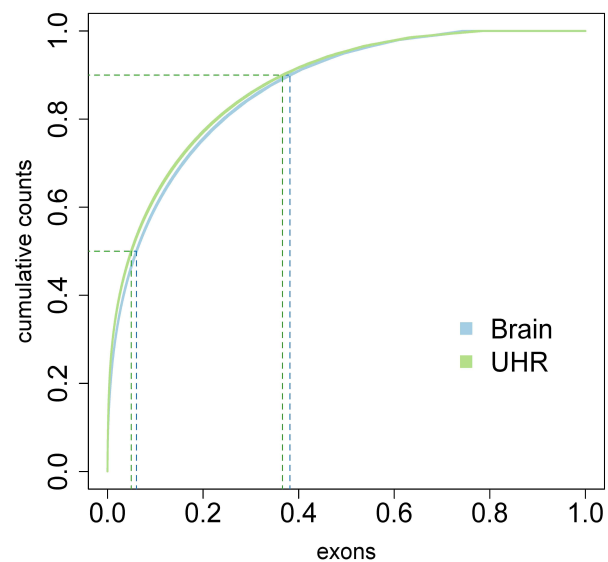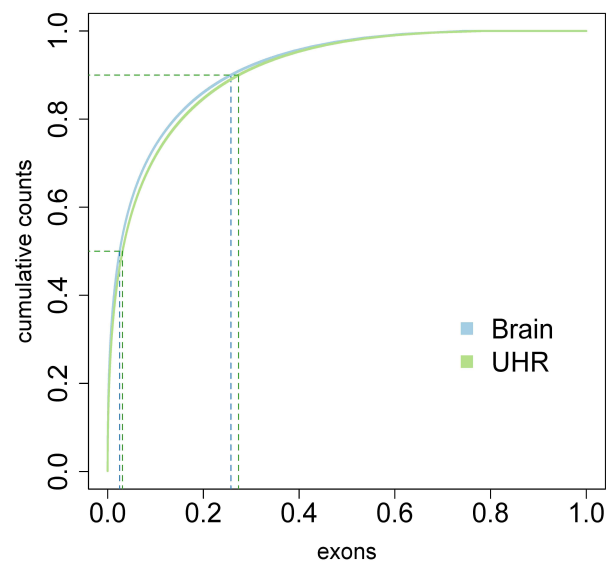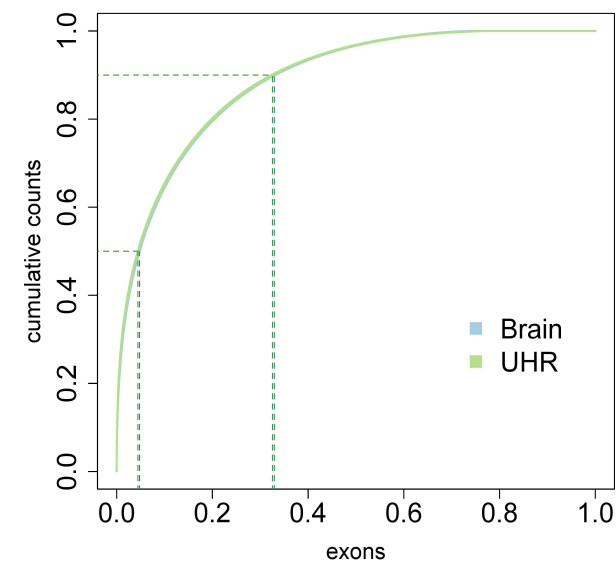

Supplement: Additional file 7 — Distribution of counts across exons. Distribution of maxcounts, totcounts and RPKM-corrected totcounts (RPKM) across exons, in Griffith's and MAQC2 data sets. Plots represent cumulative counts/RPKMs (y-axis, percentage referred to total counts/RPKMs in a library) assigned to exons (x-axis, percentage referred to the number of exons with more than zero counts/RPKMs). Each curve represents one library and different colours identify different groups. Dashed lines represent 50% and 90% of total counts/RPKMs and are summarized in Table 1. [file 1471-2105-15-S1-S7-S7.pdf]

**variance**

**coefficient of variation**

Brain

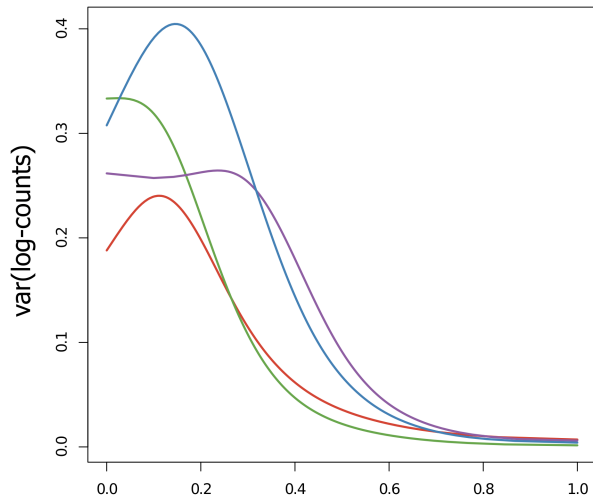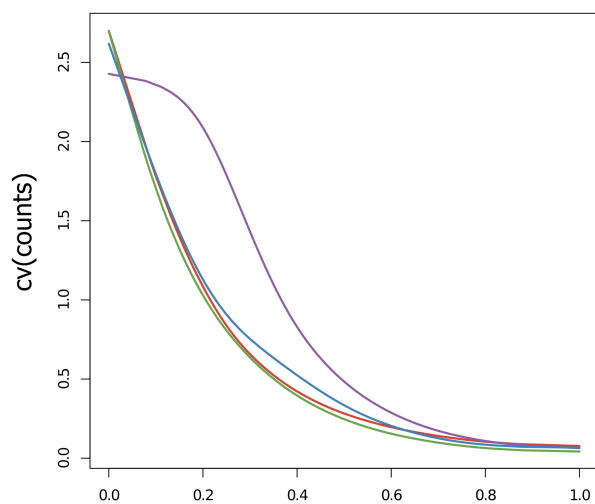

UHR

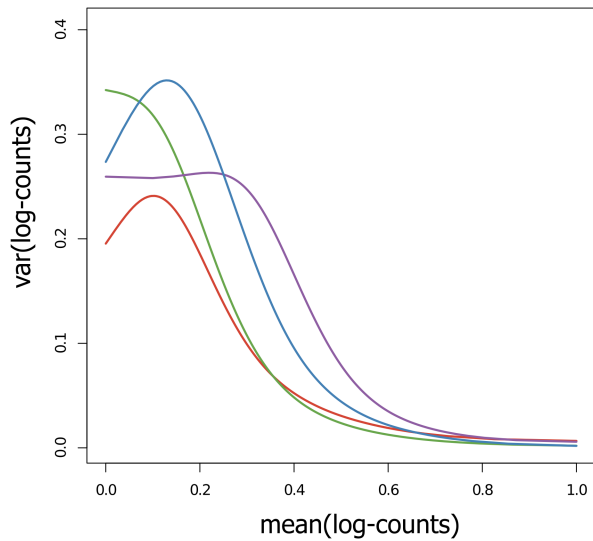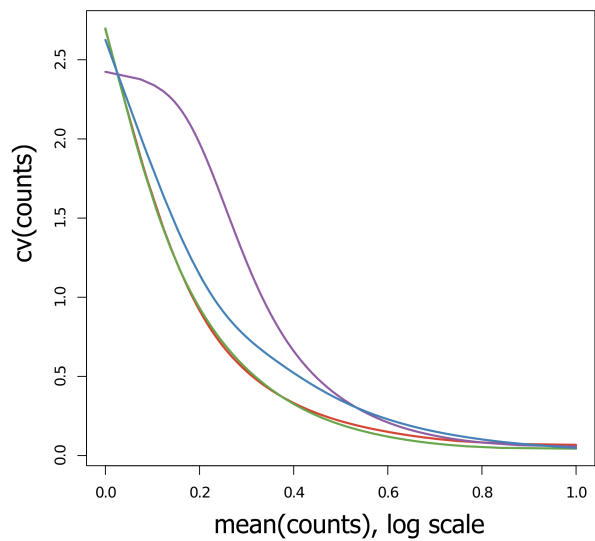

maxcounts

totcounts

RPKM

FullQ

Supplement: Additional file 9 — Data variance and coefficient of variation (MAQC2). Variance and coefficient of variation (CV) of MAQC2 data: variance vs. mean of log-counts/RPKMs (left plots) and CV vs. log-mean of counts/RPKMs (right plots). Curves represent cubic-spline fits computed on variances/CVs, averaged in bins of 5000 exons each. Since maxcounts, totcounts, and totcounts normalized with RPKM (RPKM) and within-lane full-quantile normalization over exon length (FullQ) approaches are compared, x-values are scaled to cover the range [0, 1] in order to make them comparable. [file 1471-2105-15-S1-S7-S9.pdf]

MIP101

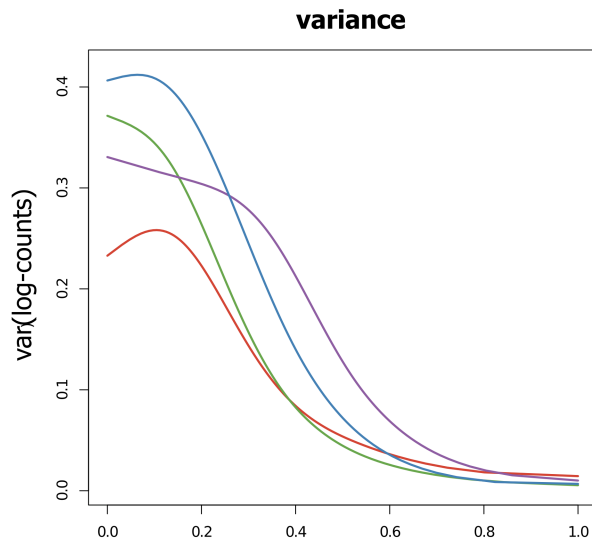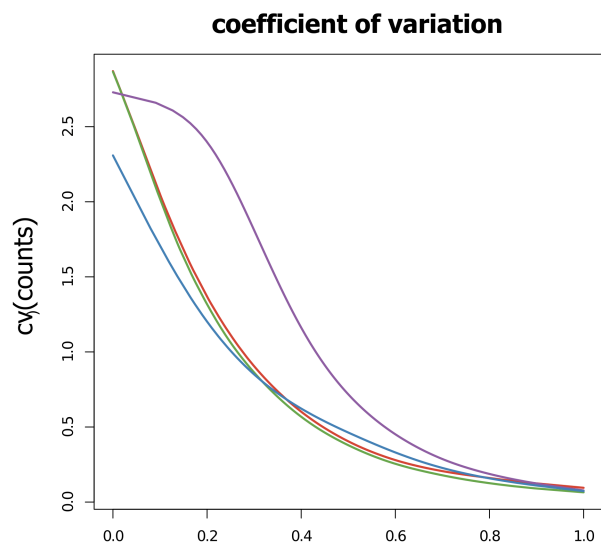

MIP5FU

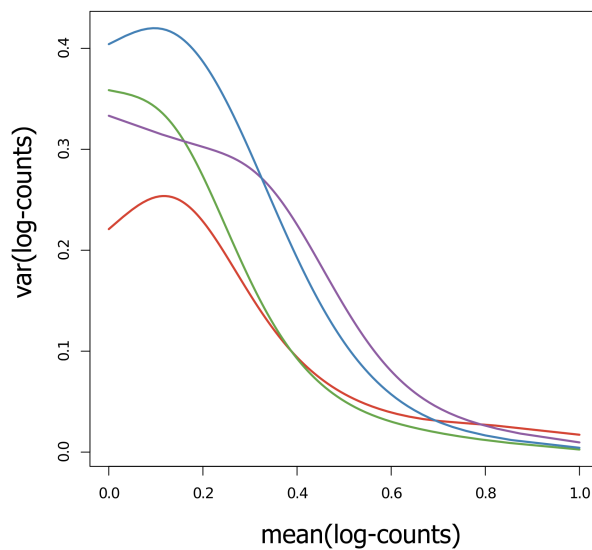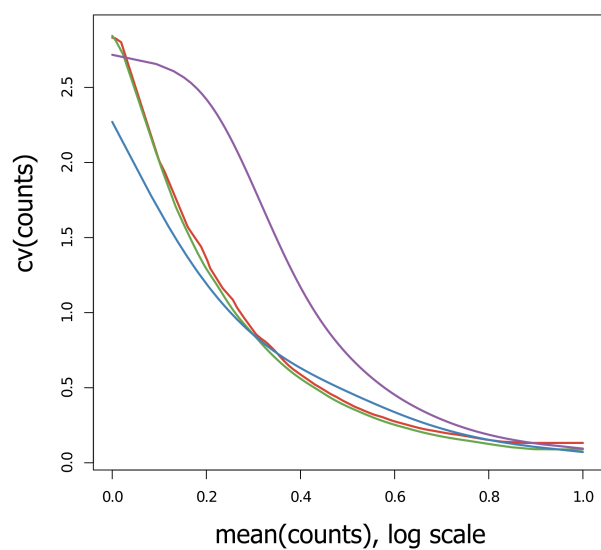

maxcounts totcounts RPKM FullQ

Supplement: Additional file 10 — Data variance and coefficient of variation (Griffith). Variance and coefficient of variation (CV) of Griffith's data: variance vs. mean of log-counts/RPKMs (left plots) and CV vs. log-mean of counts/RPKMs (right plots). Curves represent cubic-spline fits computed on variances/CVs, averaged in bins of 5000 exons each. Since maxcounts, totcounts, and totcounts normalized with RPKM (RPKM) and full-quantile (FullQ) approaches are compared, x-values are scaled to cover the range [0, 1] in order to make them comparable. [file 1471-2105-15-S1-S7-S10.pdf]

**A**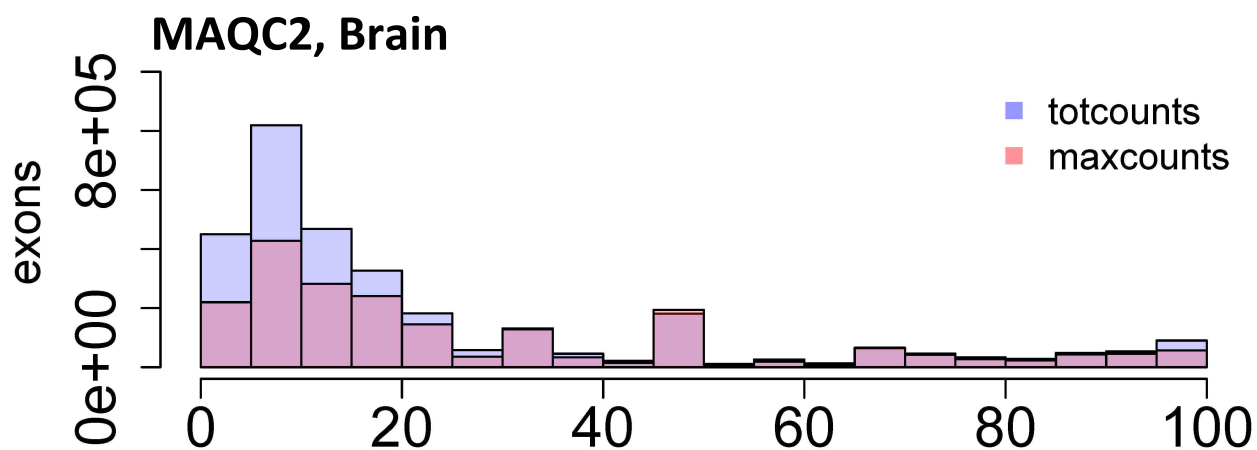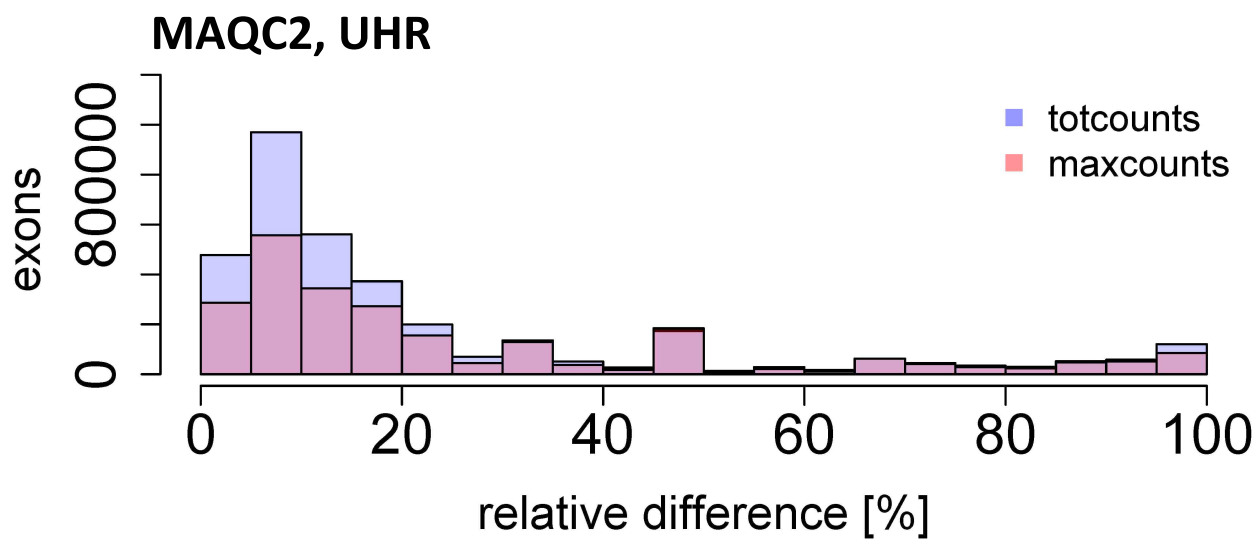**B**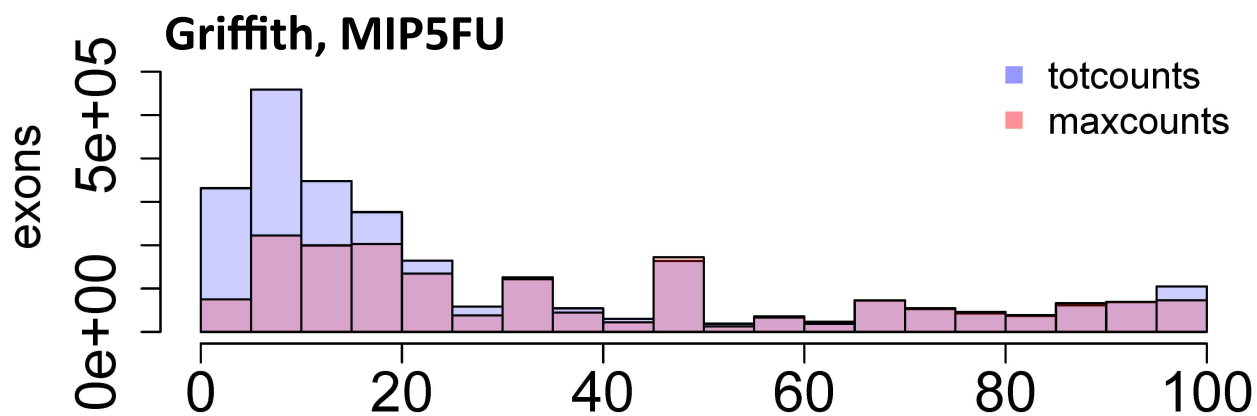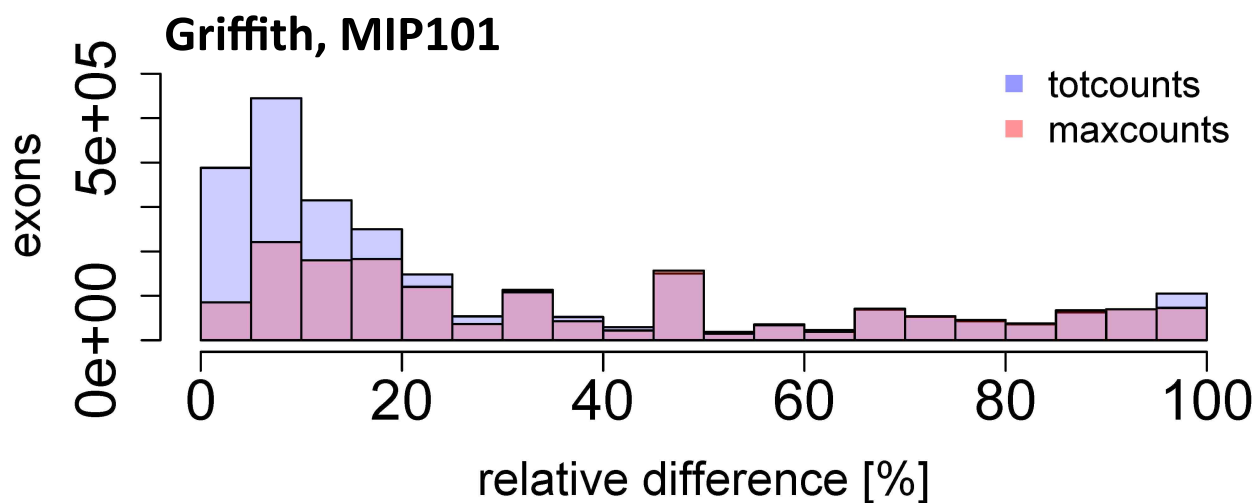

Supplement: Additional file 11 — Effect of alignment quality. Superimposed histograms of relative variation of non-normalized totcounts (blue) and maxcounts (red) when low-similarity alignments and multireads are discarded (only null-variations are reported) for MAQC2 (A) and Griffith's data (B). [file 1471-2105-15-S1-S7-S11.pdf]
