# Supplementary material for: Reducing bias in RNA sequencing data: a novel approach to compute counts
Source: BMC Bioinformatics. 2014 Jan 10;15(Suppl 1):S7. doi: 10.1186/1471-2105-15-S1-S7 (PMC4016203; doi:10.1186/1471-2105-15-S1-S7)
Supplement: Additional file 8 — Quantification of spike-in RNAs: residues. Quantification of spike-in RNAs concentrations, in all libraries of Jiang's data set, with totcounts, maxcounts, RPKM-corrected totcounts (RPKM) and totcounts corrected with within-lane full-quantile normalization over exon length (FullQ). Plots show the residues of the linear regression of counts/RPKMs over true concentrations (log-log scale), plotted against true concentrations in log scale. [file 1471-2105-15-S1-S7-S8.pdf]

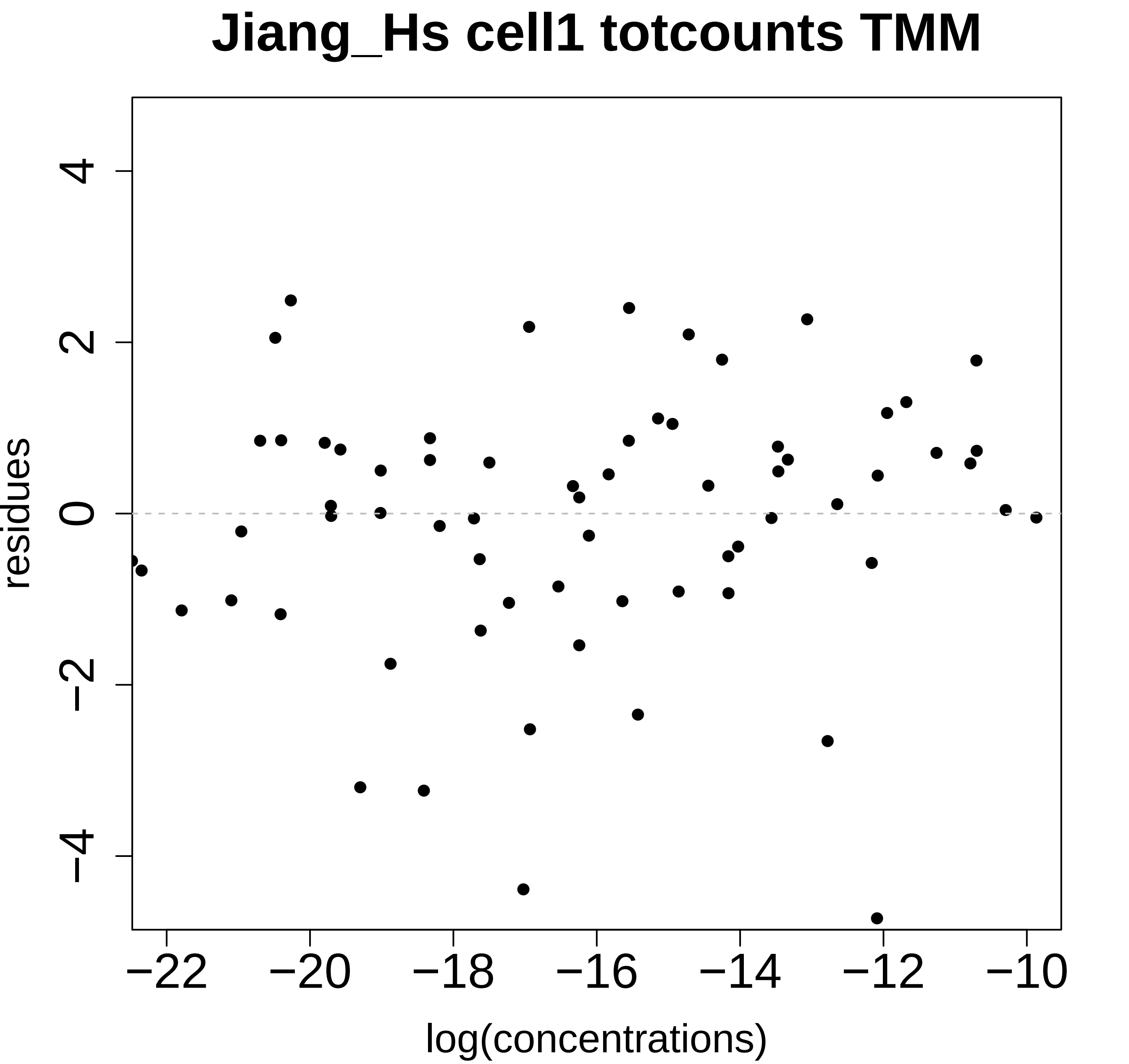
Jiang_Hs cell1 totcounts TMM
residues
log(concentrations)
4
2
0
−2
−4
−22
−20
−18
−16
−14
−12
−10

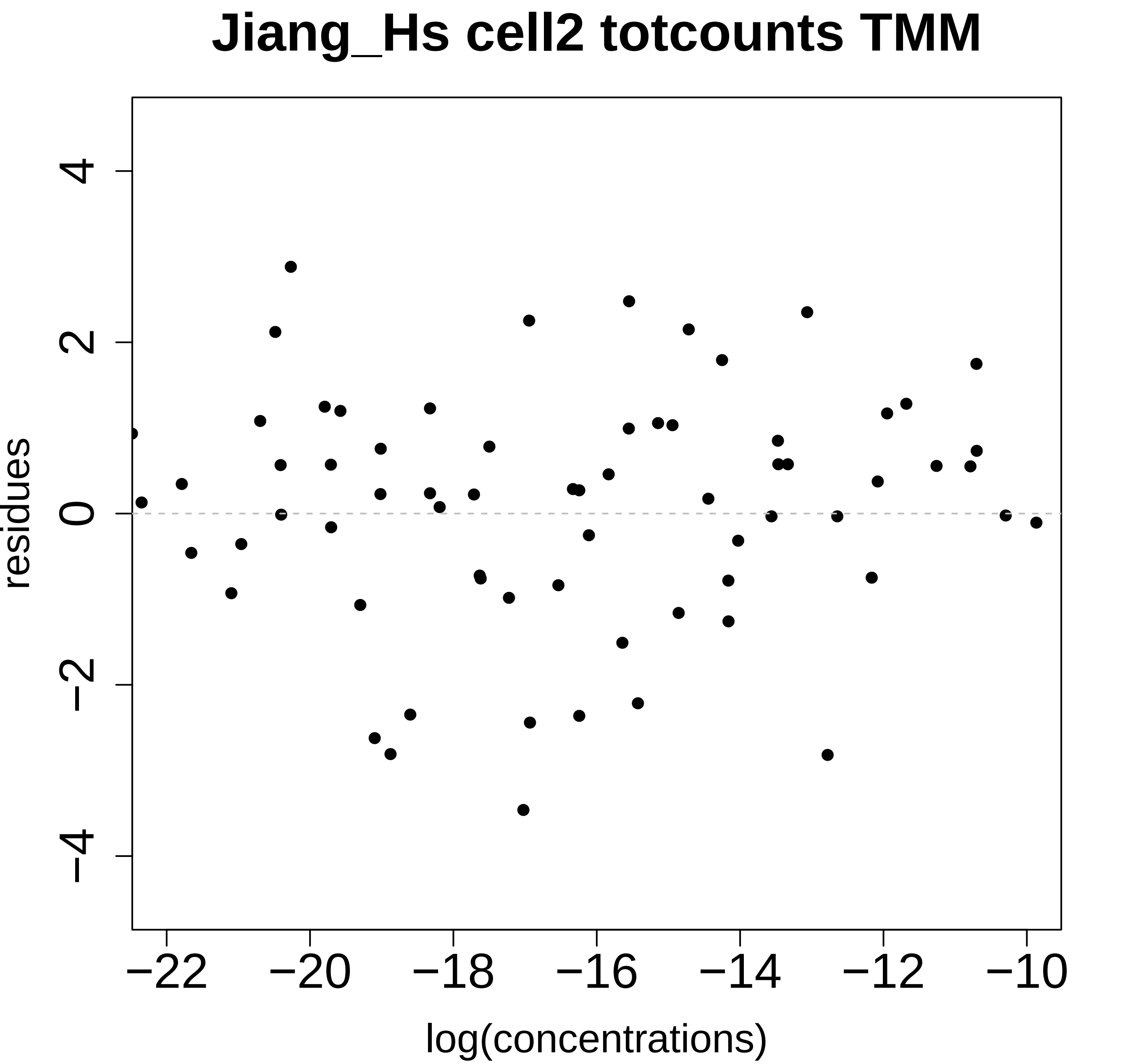
Jiang_Hs cell2 totcounts TMM
residues
4
2
0
−2
−4
−22
−20
−18
−16
−14
−12
−10
log(concentrations)

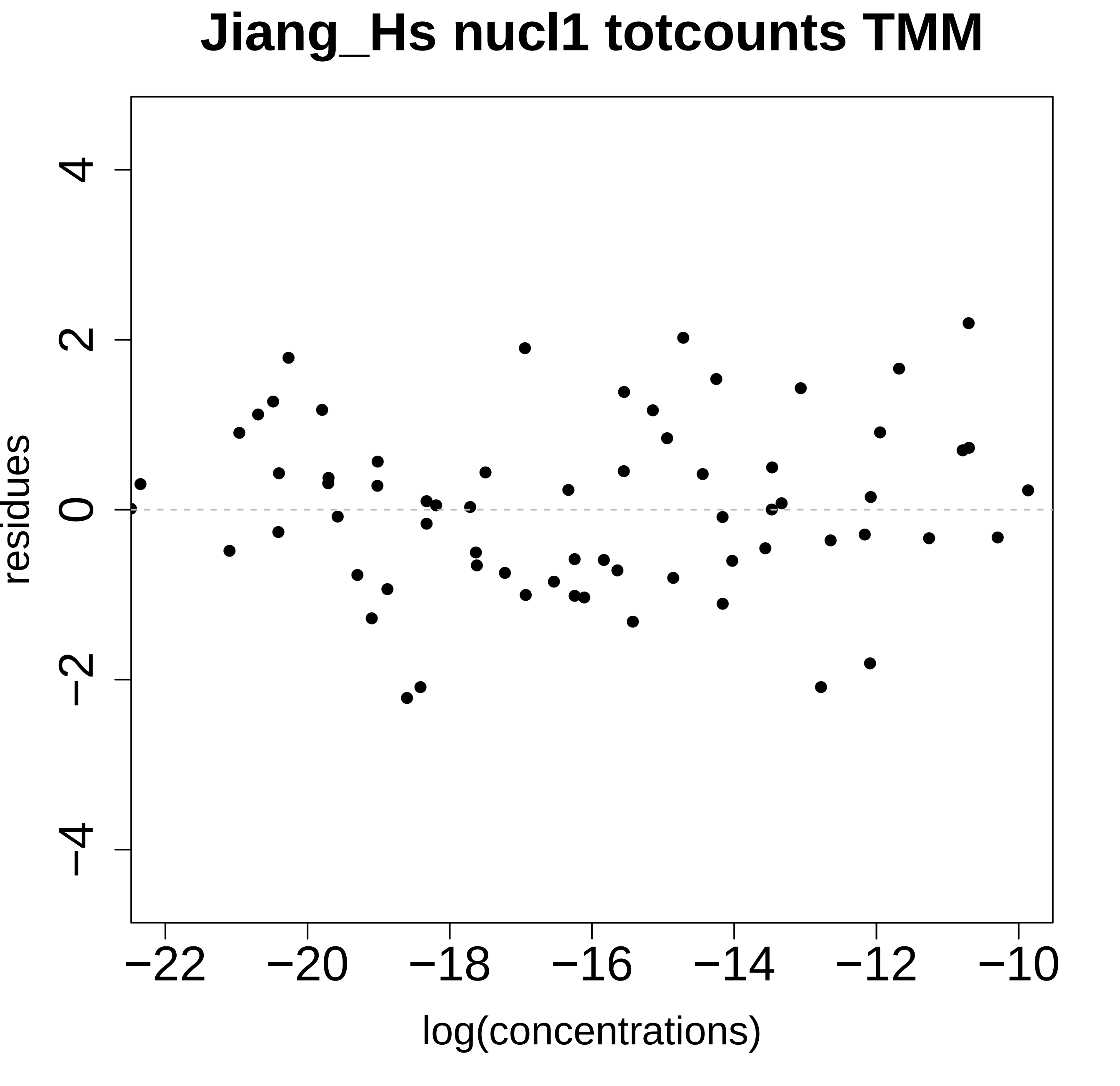

Jiang_Hs nucl1 totcounts TMM
residues
4
2
0
−2
−4
−22
−20
−18
−16
−14
−12
−10
log(concentrations)

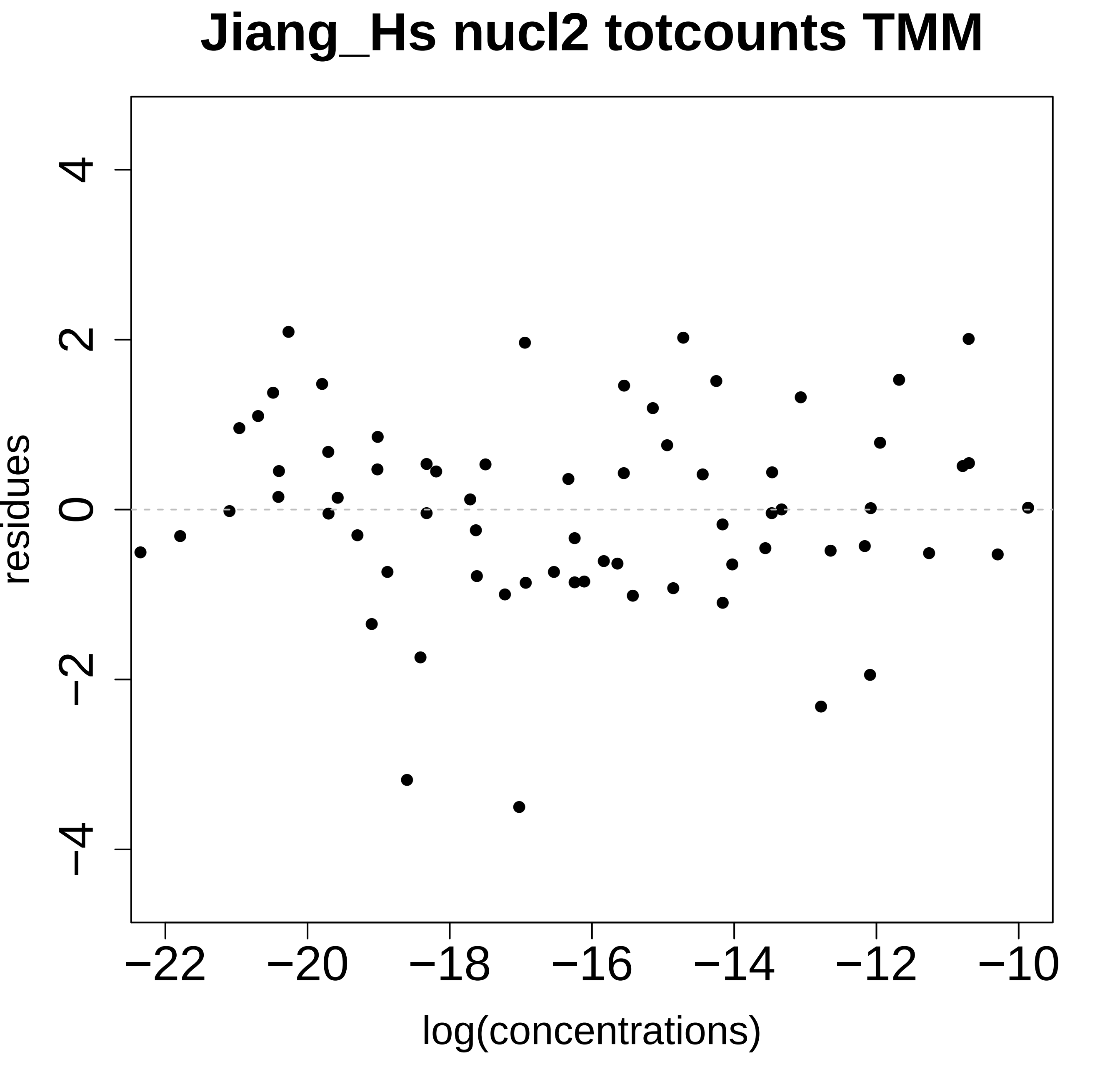

Jiang_Hs nucl2 totcounts TMM
residues
log(concentrations)
4
2
0
−2
−4
−22
−20
−18
−16
−14
−12
−10

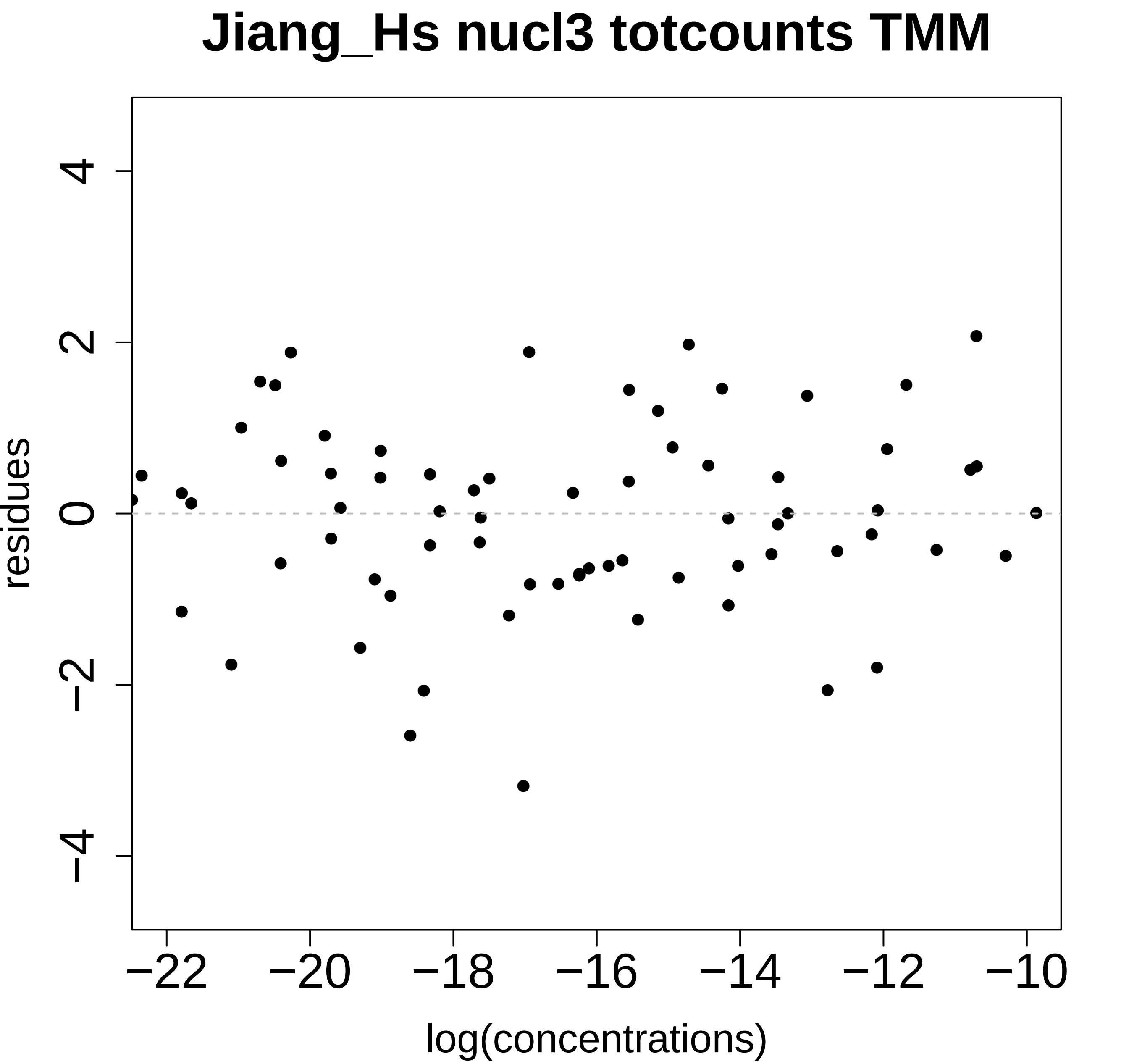
Jiang_Hs nucl3 totcounts TMM
residues
4
2
0
−2
−4
−22
−20
−18
−16
−14
−12
−10
log(concentrations)

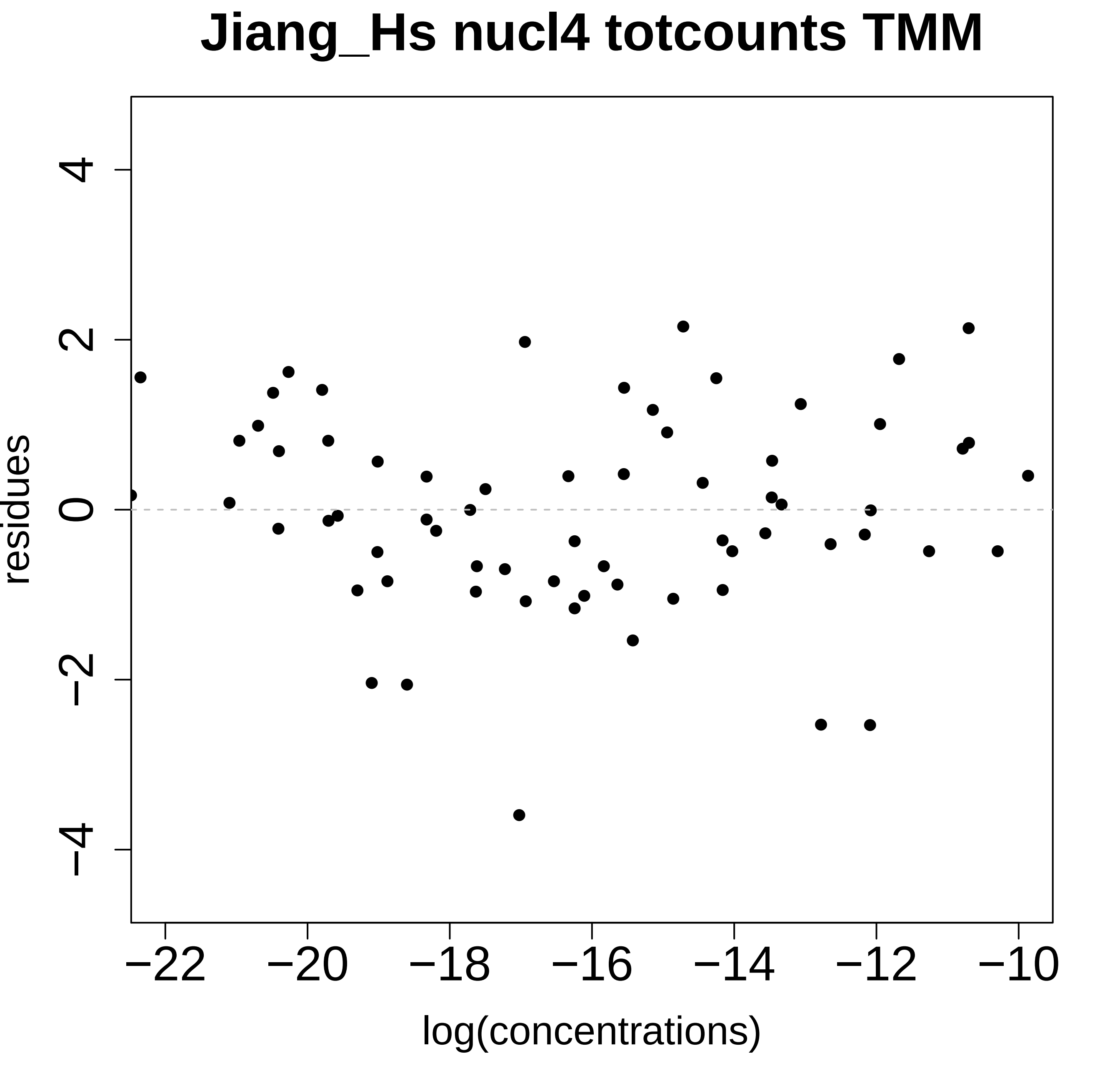
Jiang_Hs nucl4 totcounts TMM
residues
log(concentrations)
4
2
0
−2
−4
−22
−20
−18
−16
−14
−12
−10

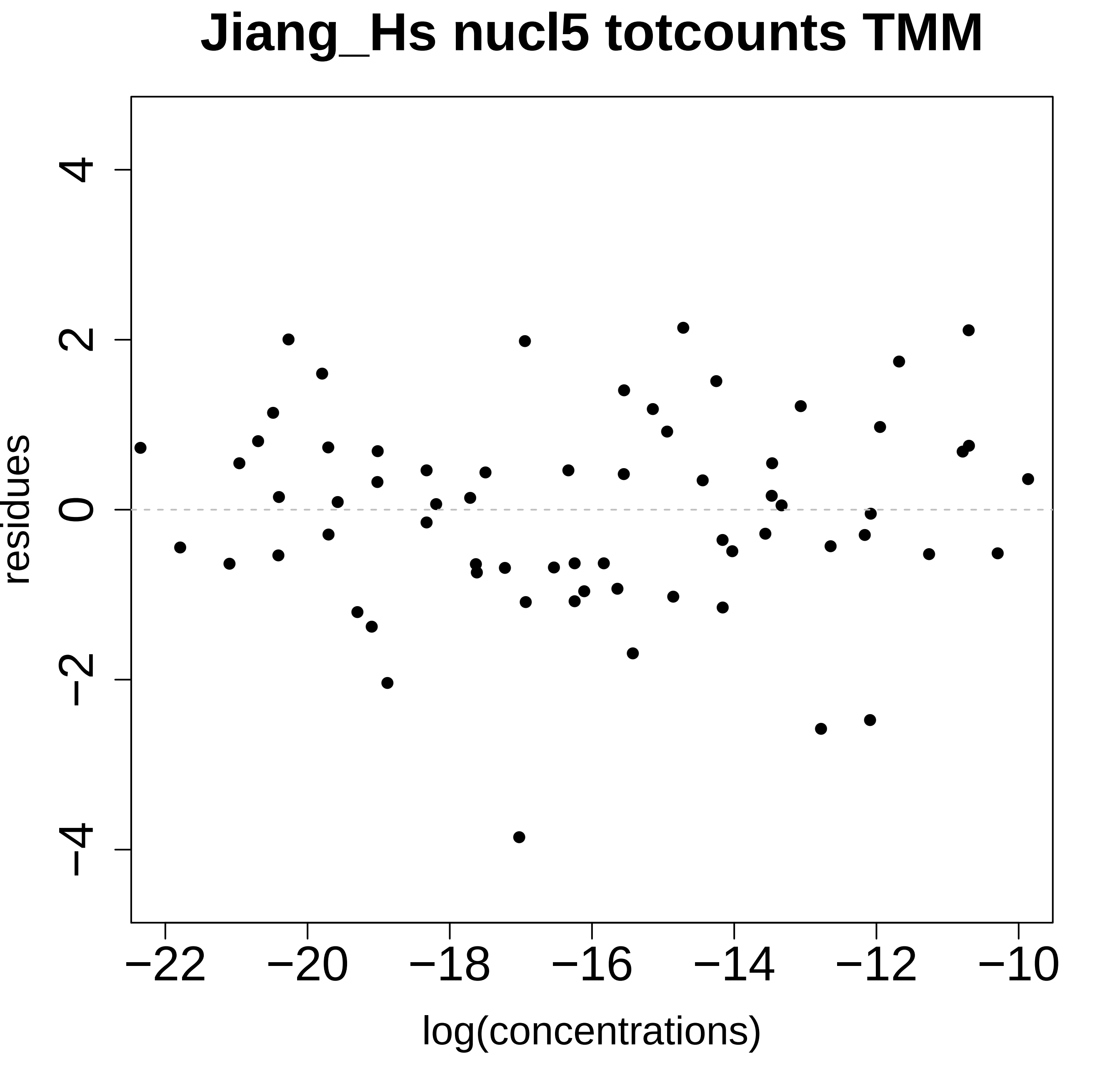

Jiang_Hs nucl5 totcounts TMM
residues
4
2
0
−2
−4
−22
−20
−18
−16
−14
−12
−10
log(concentrations)

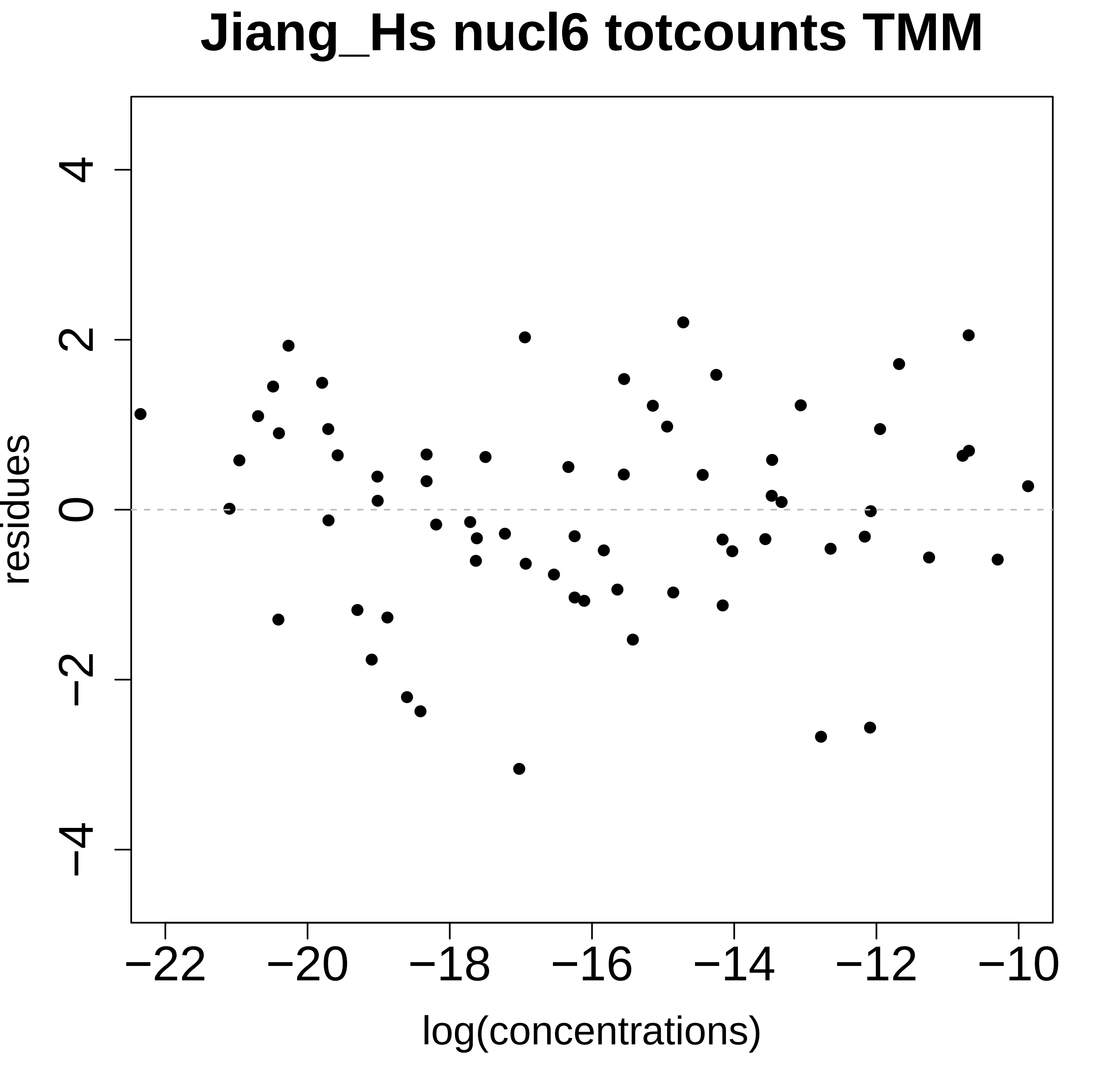
Jiang_Hs nucl6 totcounts TMM
residues
log(concentrations)
4
2
0
−2
−4
−22
−20
−18
−16
−14
−12
−10

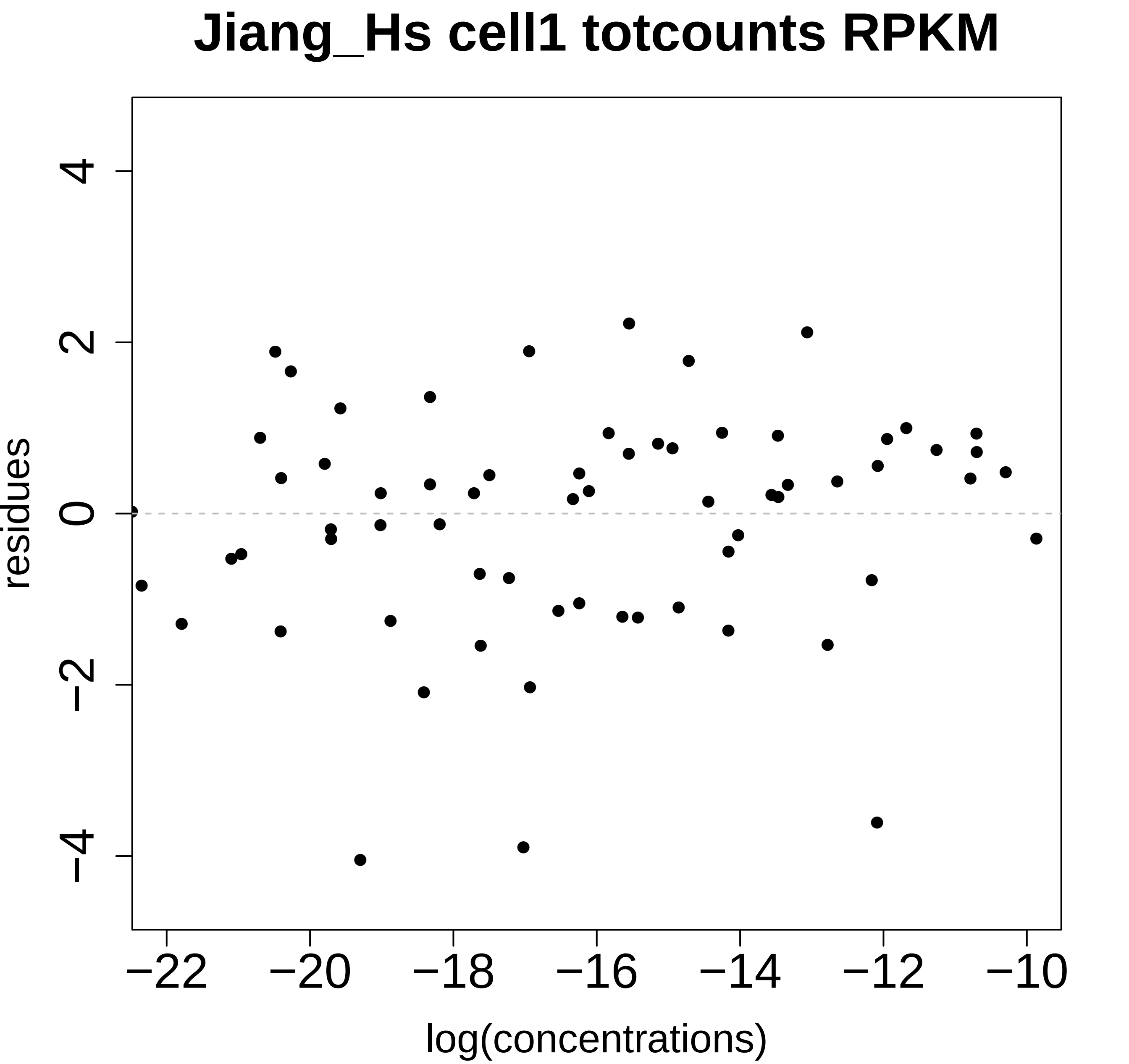
Jiang_Hs cell1 totcounts RPKM
residues
log(concentrations)
−22
−20
−18
−16
−14
−12
−10
−4
−2
0
2
4

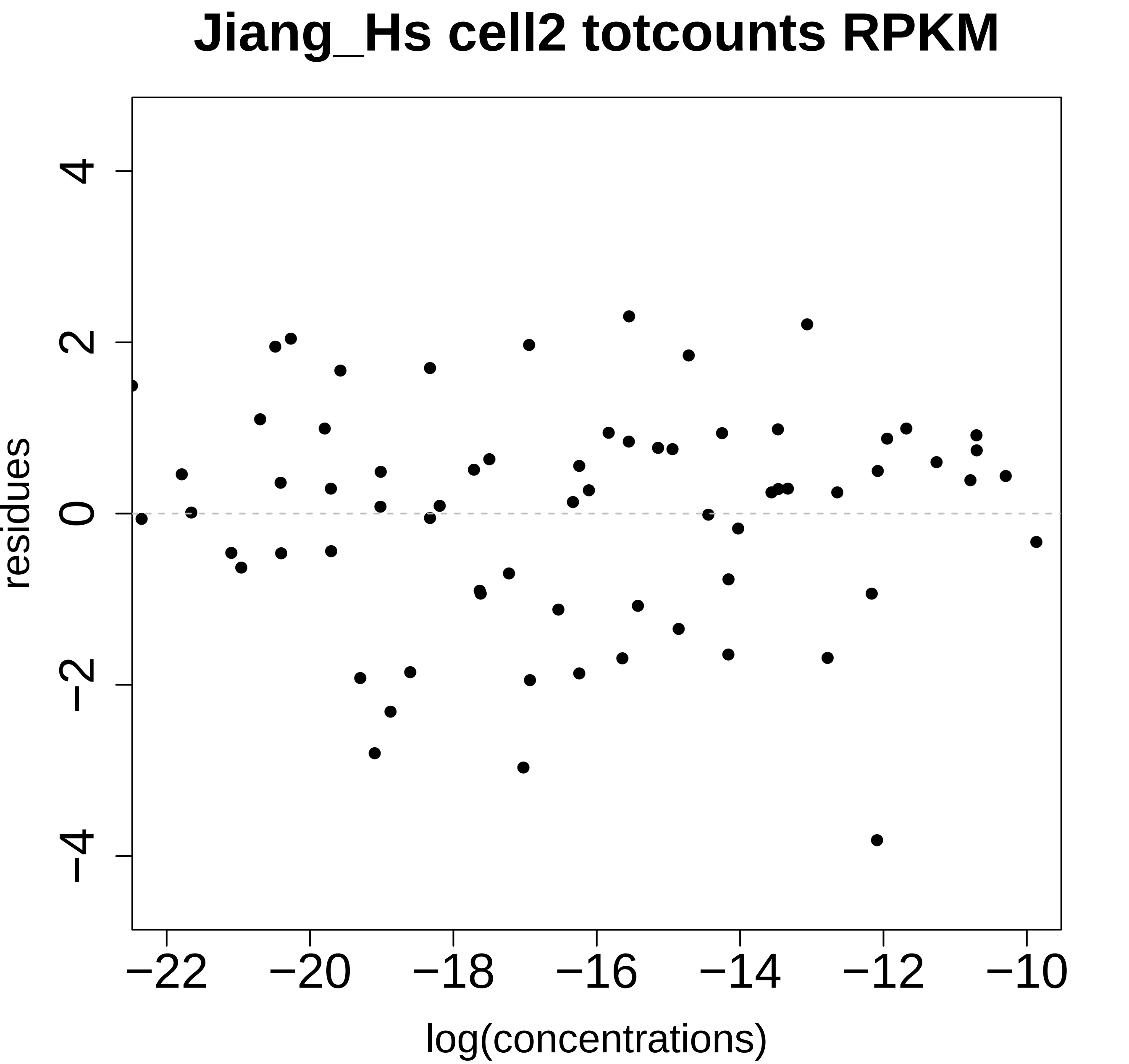

Jiang_Hs cell2 totcounts RPKM
residues
log(concentrations)
4
2
0
−2
−4
−22
−20
−18
−16
−14
−12
−10

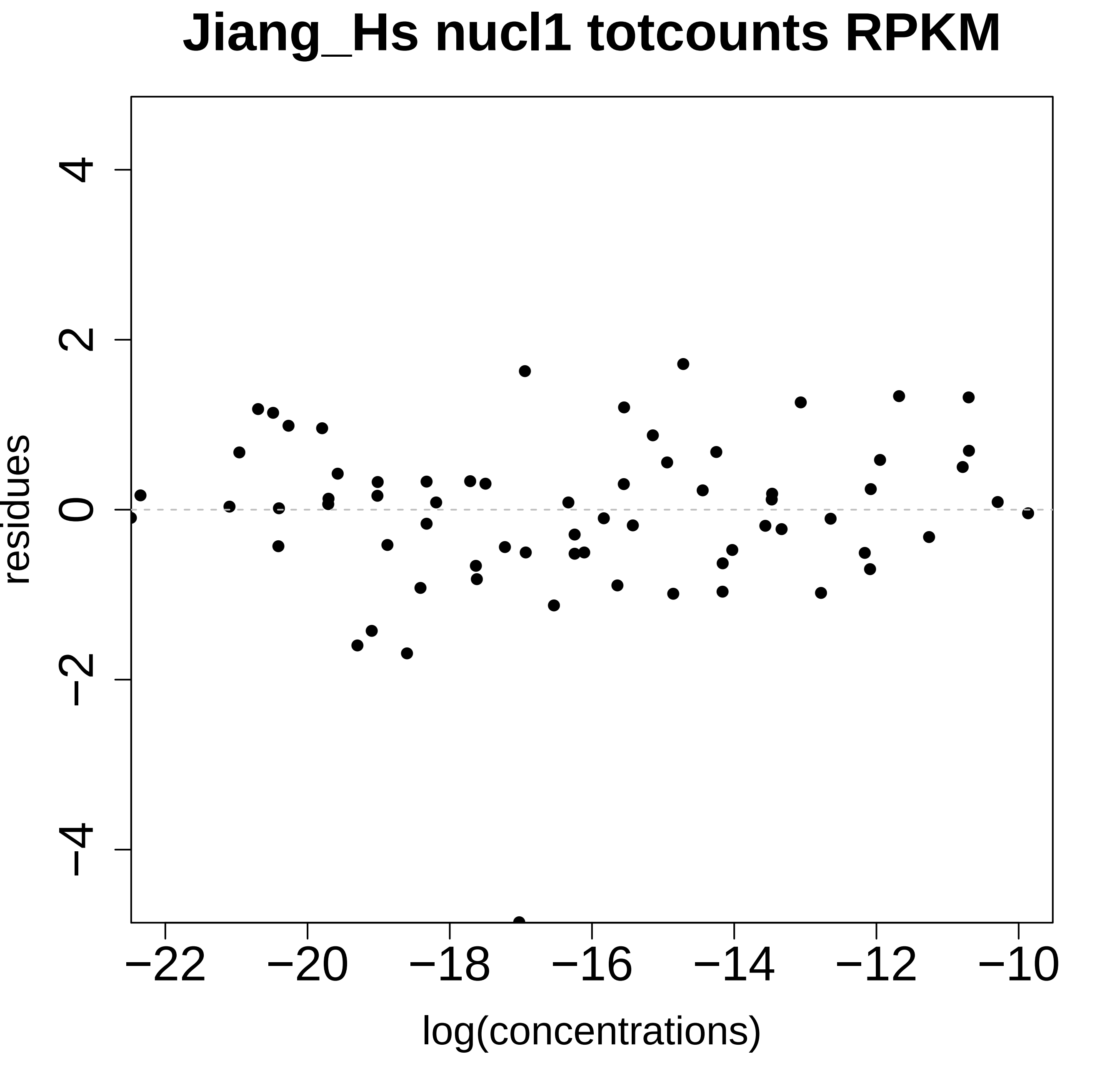

Jiang_Hs nucl1 totcounts RPKM
residues
log(concentrations)
4
2
0
−2
−4
−22
−20
−18
−16
−14
−12
−10

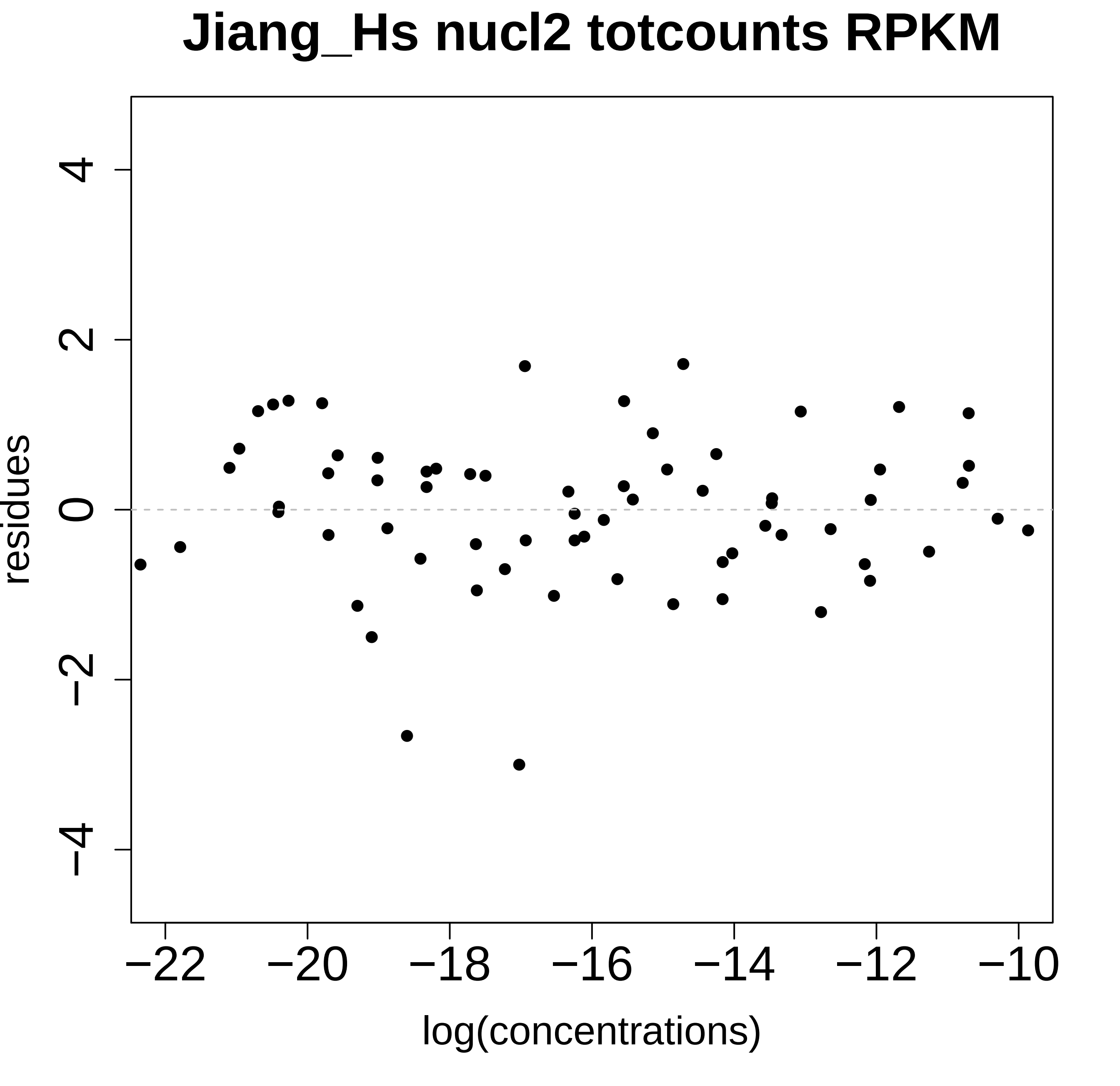

Jiang_Hs nucl2 totcounts RPKM
residues
log(concentrations)
4
2
0
−2
−4
−22
−20
−18
−16
−14
−12
−10

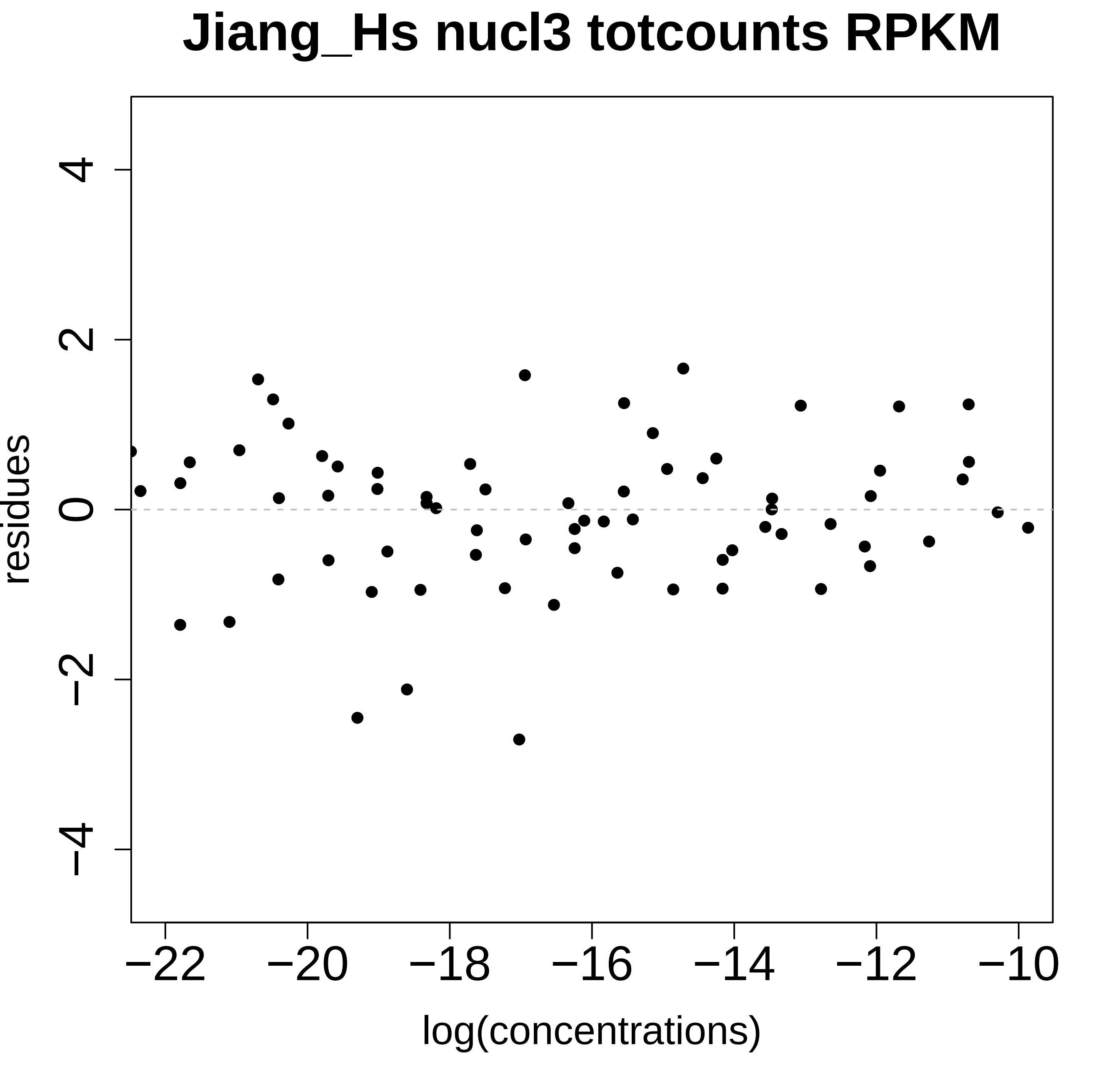

Jiang_Hs nucl3 totcounts RPKM
residues
log(concentrations)
4
2
0
−2
−4
−22
−20
−18
−16
−14
−12
−10

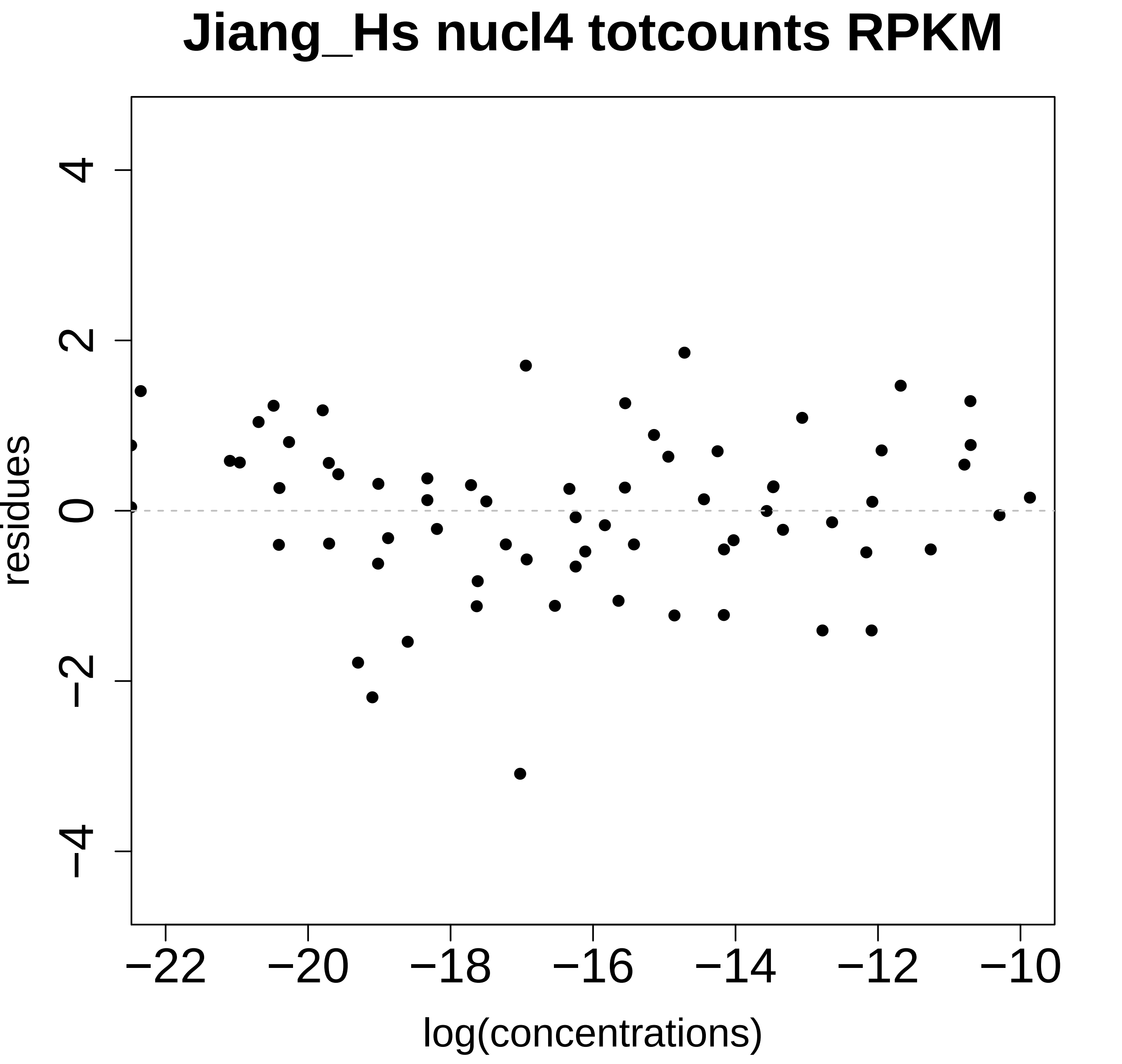

Jiang_Hs nucl4 totcounts RPKM
residues
log(concentrations)
4
2
0
−2
−4
−22
−20
−18
−16
−14
−12
−10

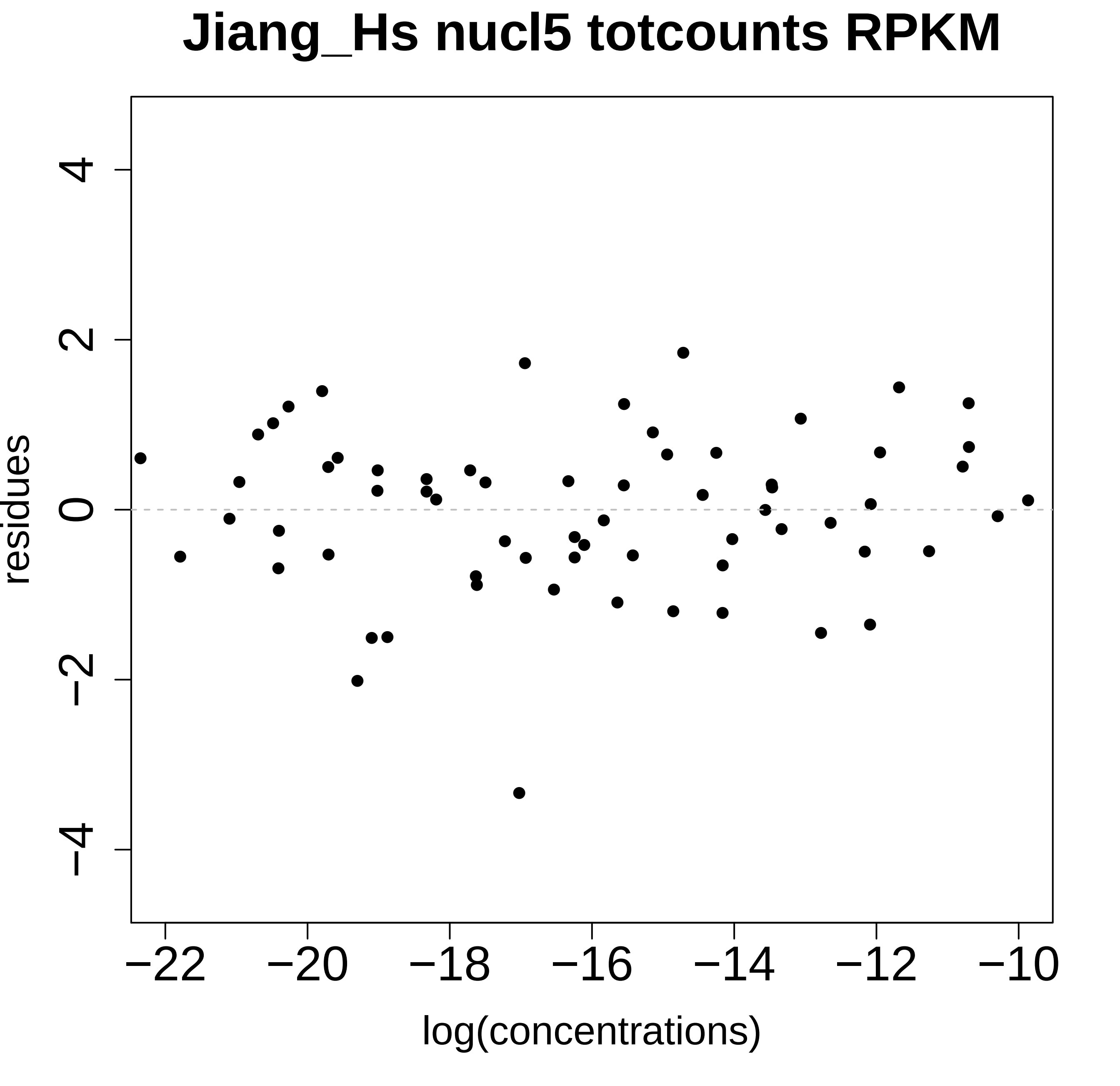

Jiang_Hs nucl5 totcounts RPKM
residues
4
2
0
−2
−4
−22
−20
−18
−16
−14
−12
−10
log(concentrations)

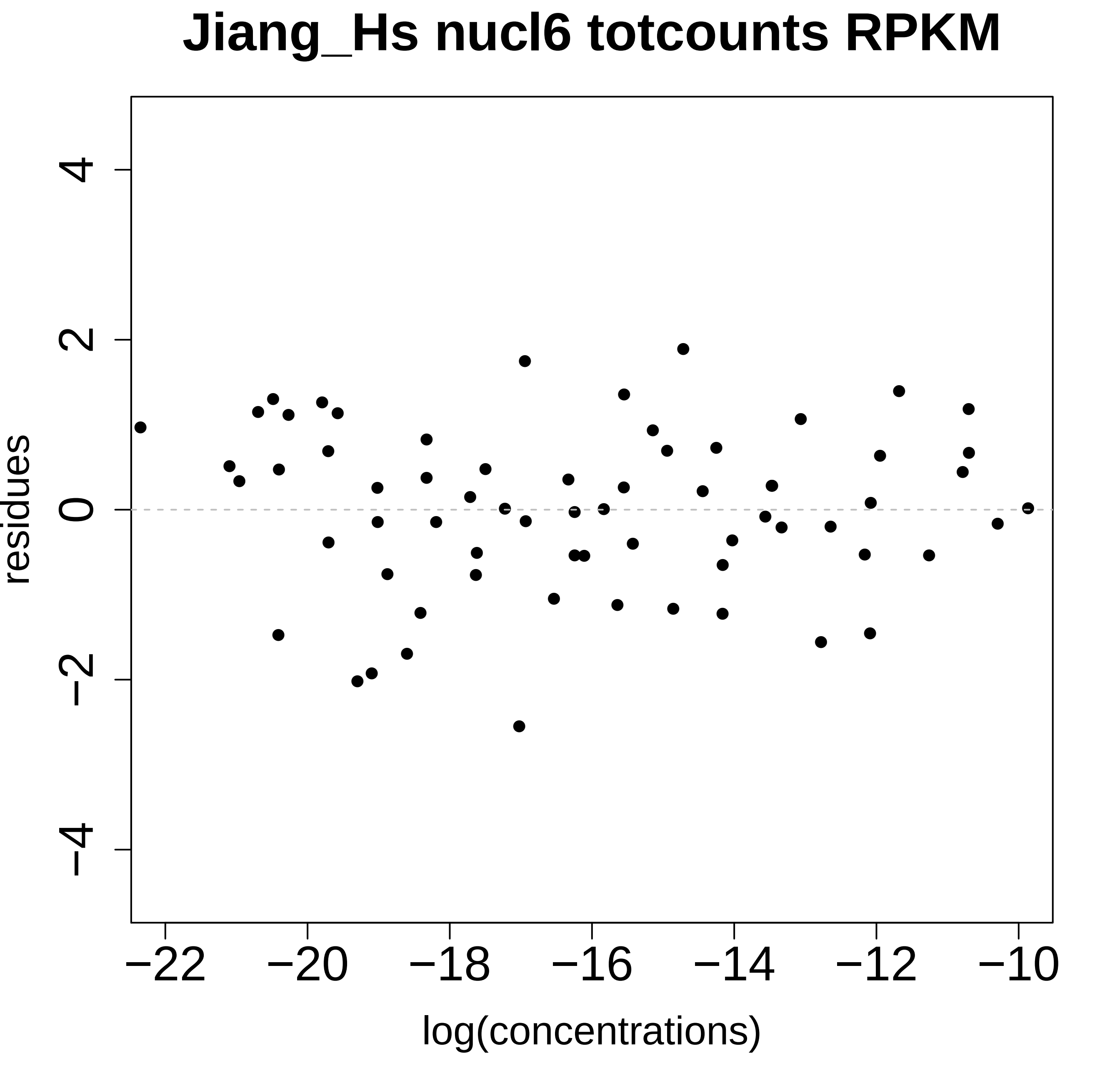

Jiang_Hs nucl6 totcounts RPKM
residues
log(concentrations)
4
2
0
−2
−4
−22
−20
−18
−16
−14
−12
−10

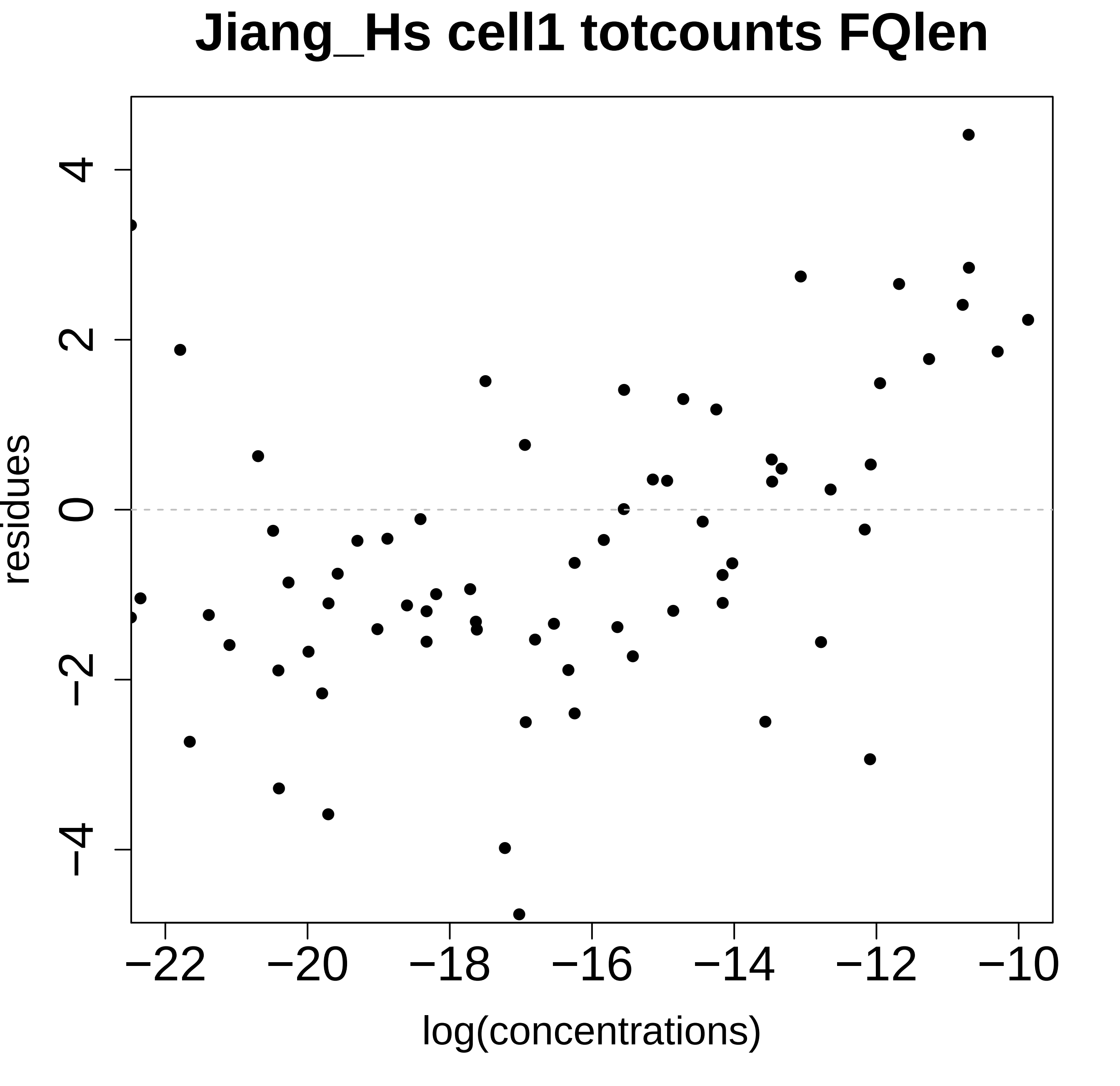

Jiang_Hs cell1 totcounts FQlen
residues
4
2
0
−2
−4
−22
−20
−18
−16
−14
−12
−10
log(concentrations)

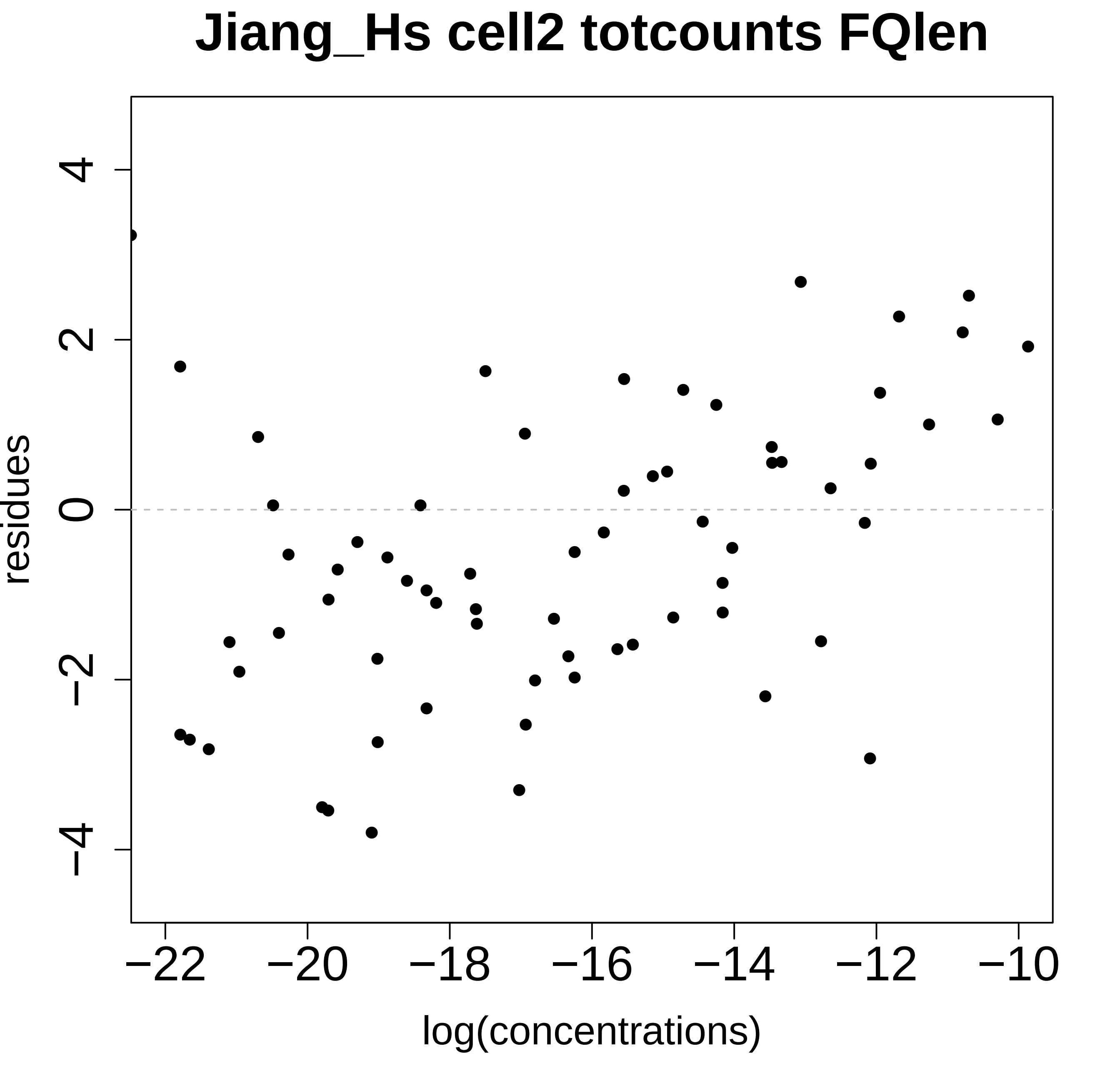

Jiang_Hs cell2 totcounts FQlen
residues
log(concentrations)
4
2
0
−2
−4
−22
−20
−18
−16
−14
−12
−10

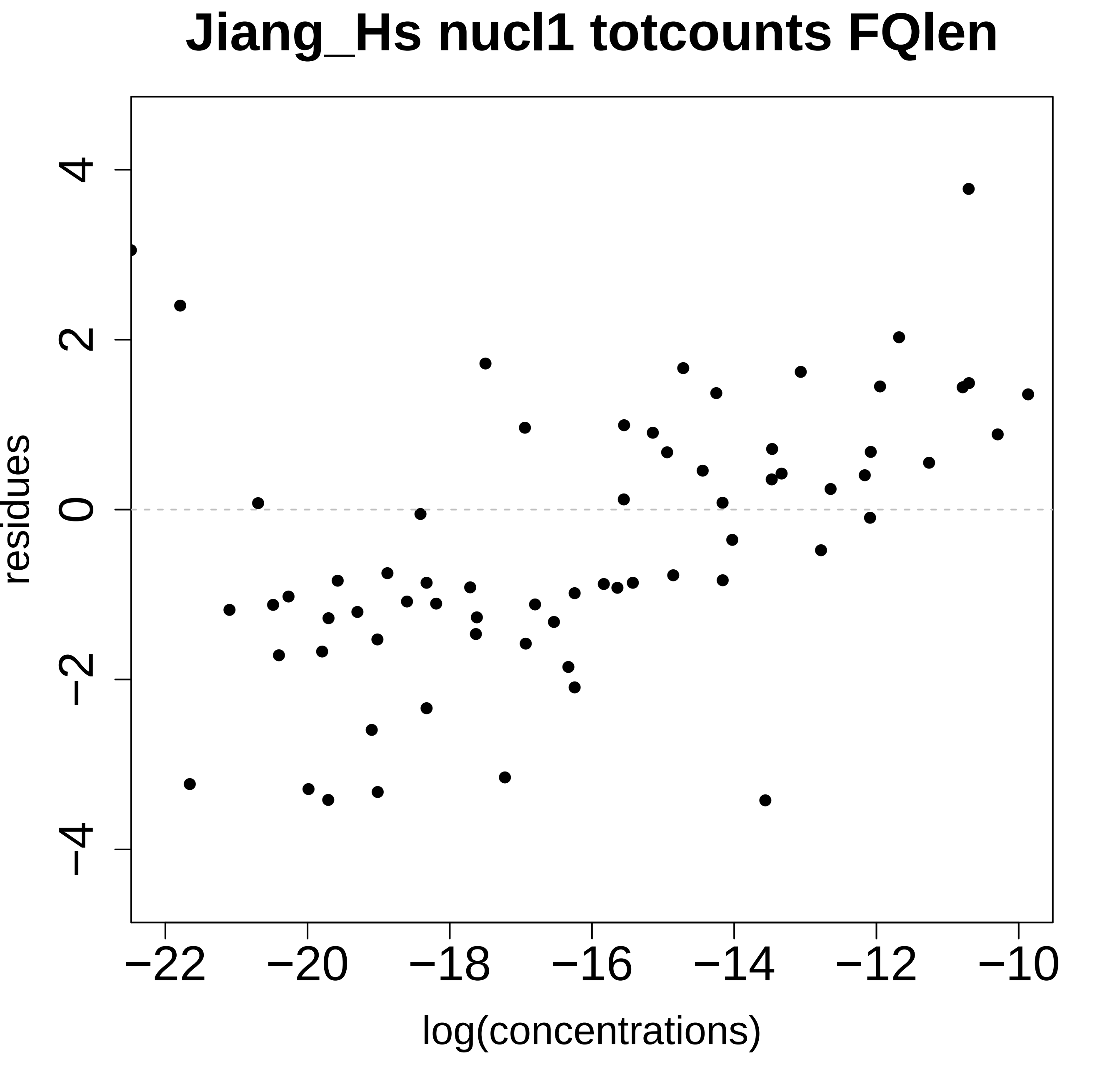
Jiang_Hs nucl1 totcounts FQlen
residues
log(concentrations)
4
2
0
−2
−4
−22
−20
−18
−16
−14
−12
−10

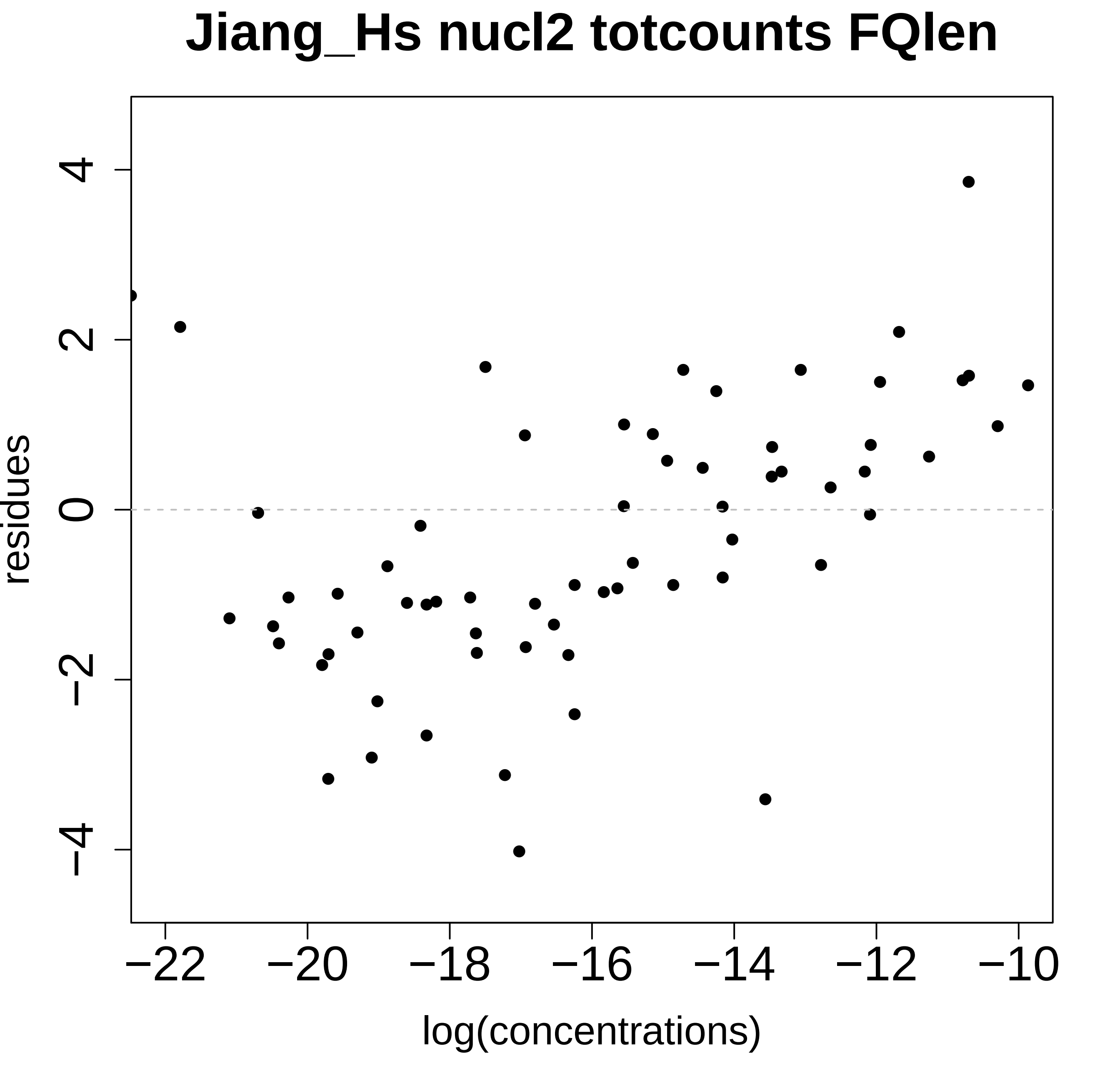

Jiang_Hs nucl2 totcounts FQlen
residues
log(concentrations)
4
2
0
−2
−4
−22
−20
−18
−16
−14
−12
−10

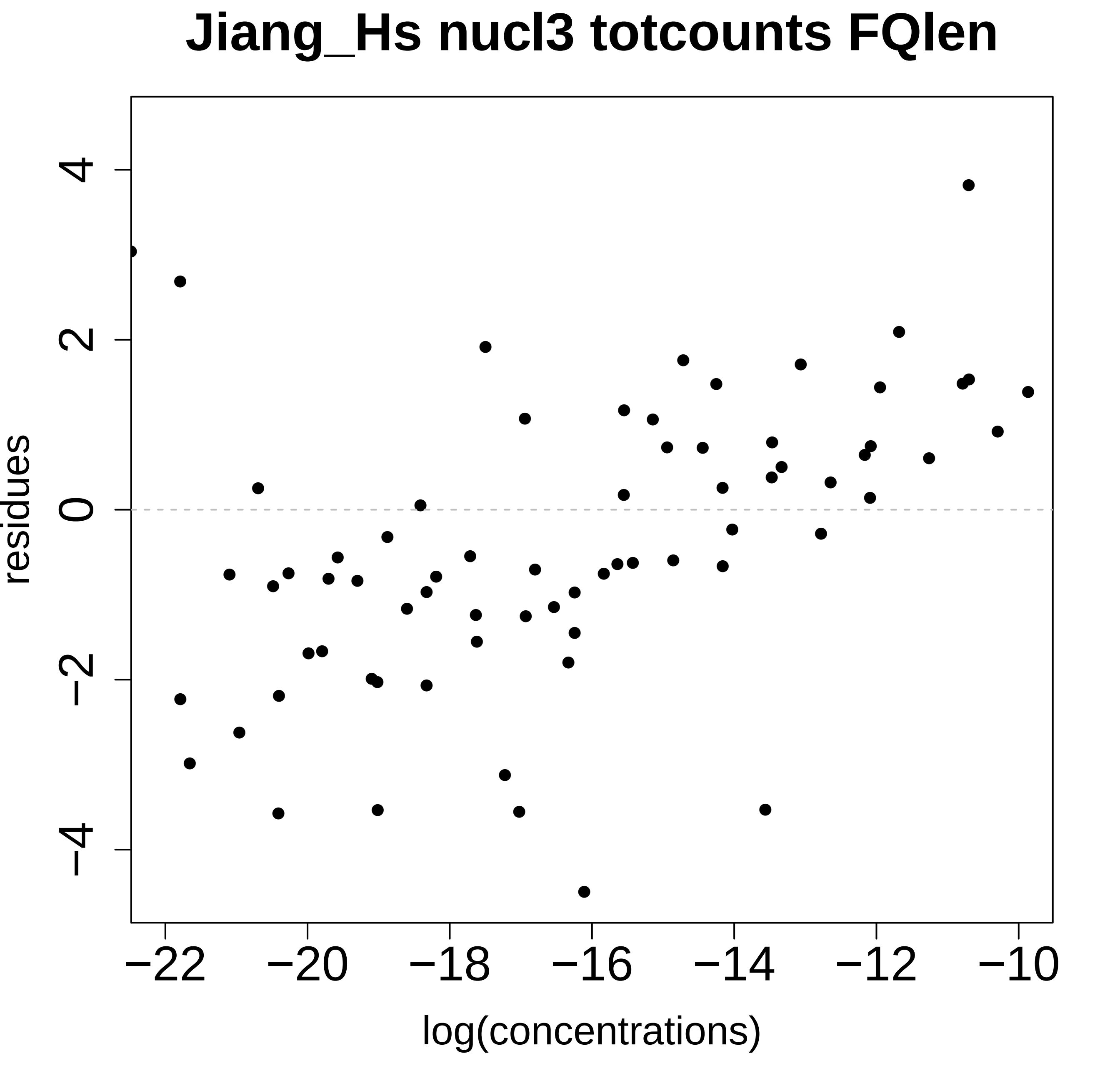
Jiang_Hs nucl3 totcounts FQlen
residues
log(concentrations)
4
2
0
−2
−4
−22
−20
−18
−16
−14
−12
−10

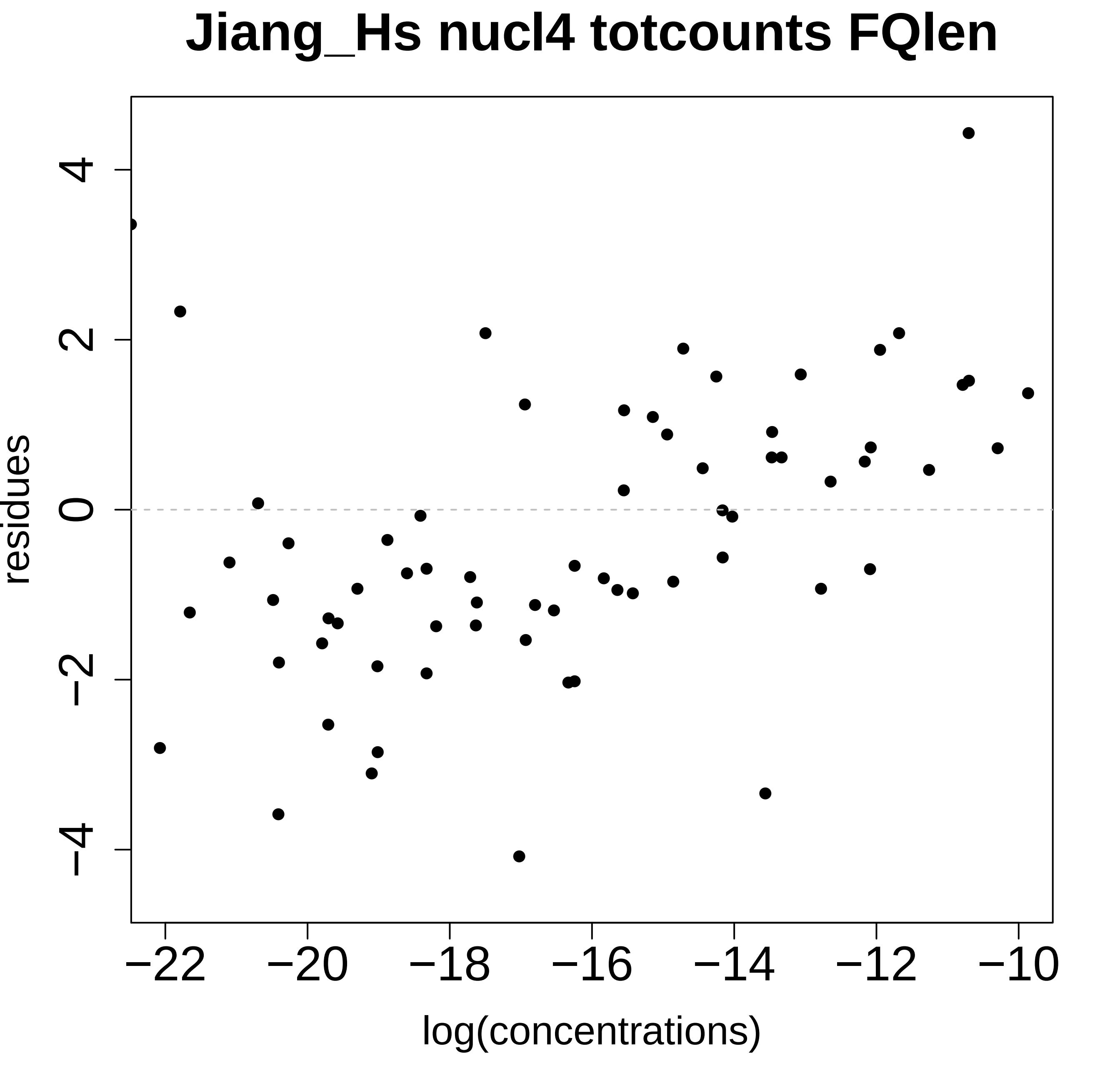
Jiang_Hs nucl4 totcounts FQlen
residues
log(concentrations)
4
2
0
−2
−4
−22
−20
−18
−16
−14
−12
−10

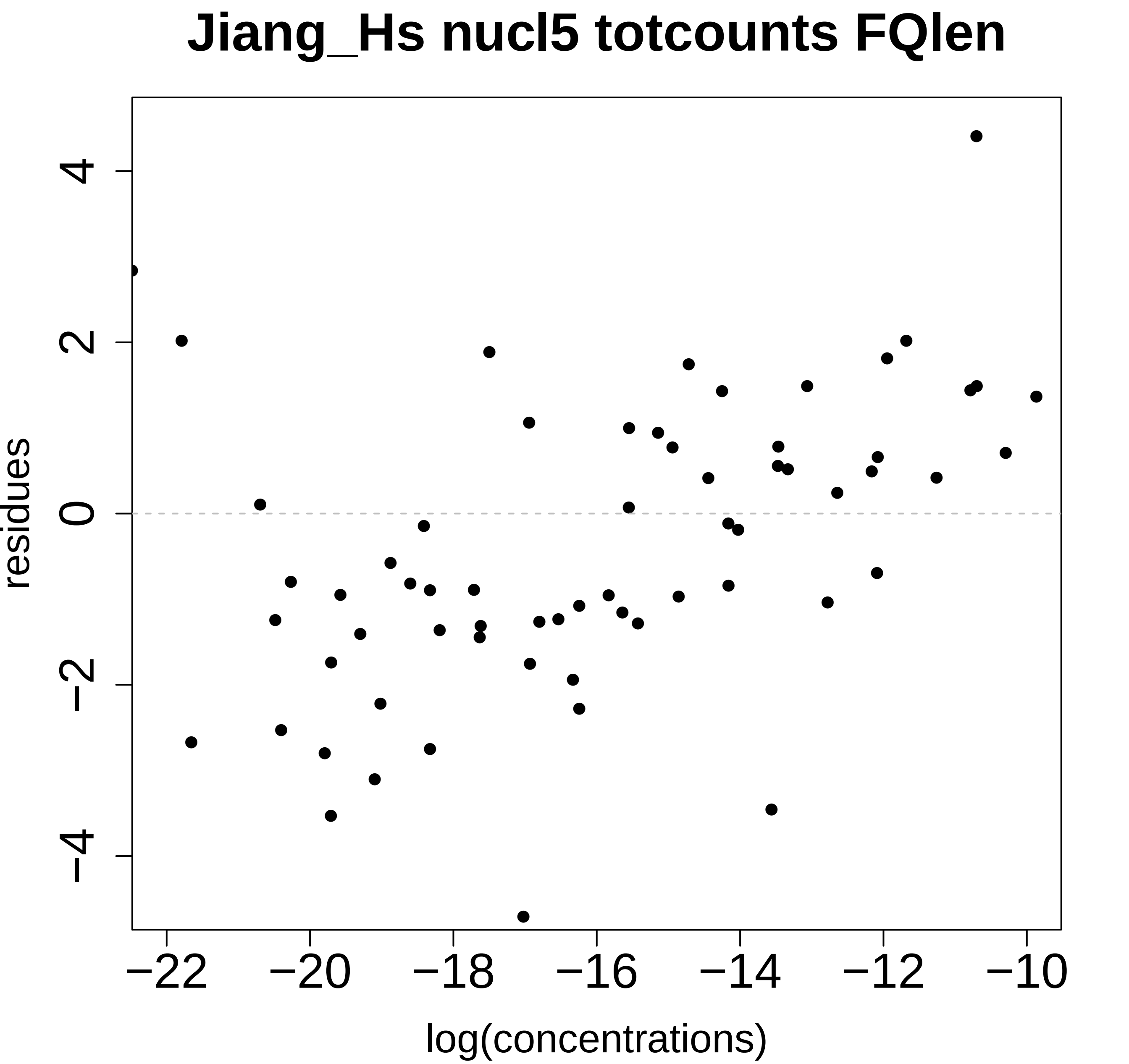
Jiang_Hs nucl5 totcounts FQlen
residues
log(concentrations)
4
2
0
−2
−4
−22
−20
−18
−16
−14
−12
−10

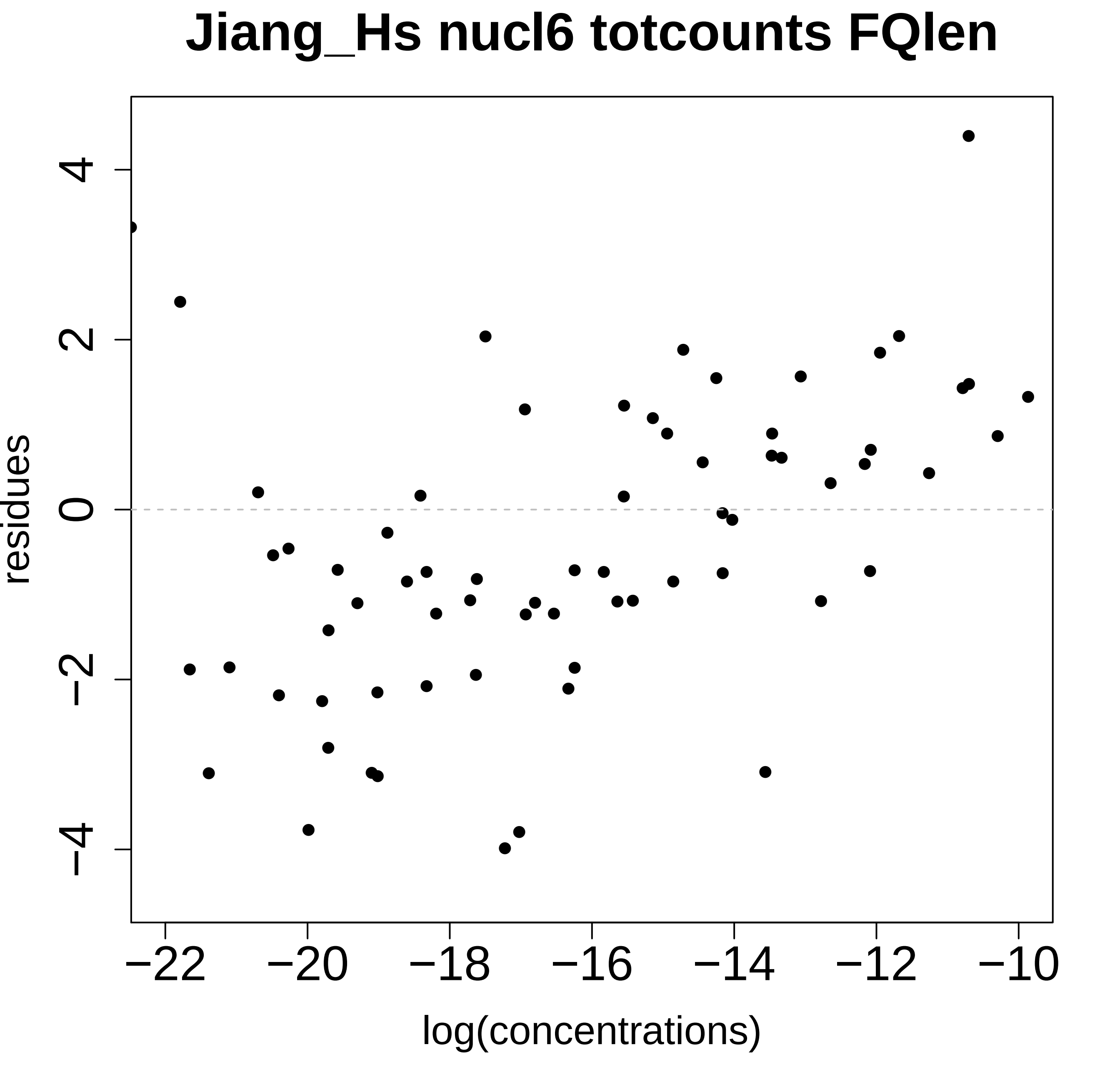
Jiang_Hs nucl6 totcounts FQlen
residues
log(concentrations)
4
2
0
−2
−4
−22
−20
−18
−16
−14
−12
−10
